# Mechanisms of immune modulation in the tumor microenvironment and implications for targeted therapy

**DOI:** 10.3389/fonc.2023.1200646

**Published:** 2023-06-22

**Authors:** Paulina Czajka-Francuz, Maria J. Prendes, Arun Mankan, Ángela Quintana, Sarabjot Pabla, Shakti Ramkissoon, Taylor J. Jensen, Sandra Peiró, Eric A. Severson, Bhagelu R. Achyut, Laura Vidal, Martine Poelman, Kamal S. Saini

**Affiliations:** ^1^ Fortrea, Inc., Durham, NC, United States; ^2^ Labcorp Oncology, Durham, NC, United States; ^3^ Breast Cancer Unit, Vall d'Hebrón Institute of Oncology, Barcelona, Spain; ^4^ Addenbrooke’s Hospital, Cambridge University Hospitals National Health Service (NHS) Foundation Trust, Cambridge, United Kingdom

**Keywords:** tumor microenvironment, immune escape mechanisms, immunotherapy, cancer, immunosuppression mechanisms

## Abstract

The efficacy of cancer therapies is limited to a great extent by immunosuppressive mechanisms within the tumor microenvironment (TME). Numerous immune escape mechanisms have been identified. These include not only processes associated with tumor, immune or stromal cells, but also humoral, metabolic, genetic and epigenetic factors within the TME. The identification of immune escape mechanisms has enabled the development of small molecules, nanomedicines, immune checkpoint inhibitors, adoptive cell and epigenetic therapies that can reprogram the TME and shift the host immune response towards promoting an antitumor effect. These approaches have translated into series of breakthroughs in cancer therapies, some of which have already been implemented in clinical practice. In the present article the authors provide an overview of some of the most important mechanisms of immunosuppression within the TME and the implications for targeted therapies against different cancers.

## 1 Introduction

Tumor growth depends to a great extent on the tumor microenvironment (TME) and the complex interactions between stromal, immune, and tumor cells. Growing evidence points to the significance of immune cell infiltration in response, prognosis (1) and TME characterization (2). The latest advances in therapies based on utilizing the host immune response has led to the development of new platforms to evaluate the immune status in tumors. Omniseq INSIGHT, as an example, is a next-generation sequencing technology utlizing DNA and RNA sequencing to determine the mutational status of solid tumors and their immune-phenotype. These assessments enable the identification of potential treatment options for patients.

The presence of pre-existing immunity, defined as the infiltration of immune cells into the tumor, seems to be crucial for the response to immunotherapies (3). Based on the histopathological localization of CD8 cytotoxic T lymphocytes (CTLs) within the tumor, three categories of TME have been proposed: (1) hot (inflamed) TME with pre-existing immunity, (2) immunologically excluded TME (intermediate stage), and (3) cold TME (non-inflamed, immunologically ignorant) (4).

**Figure 1:**
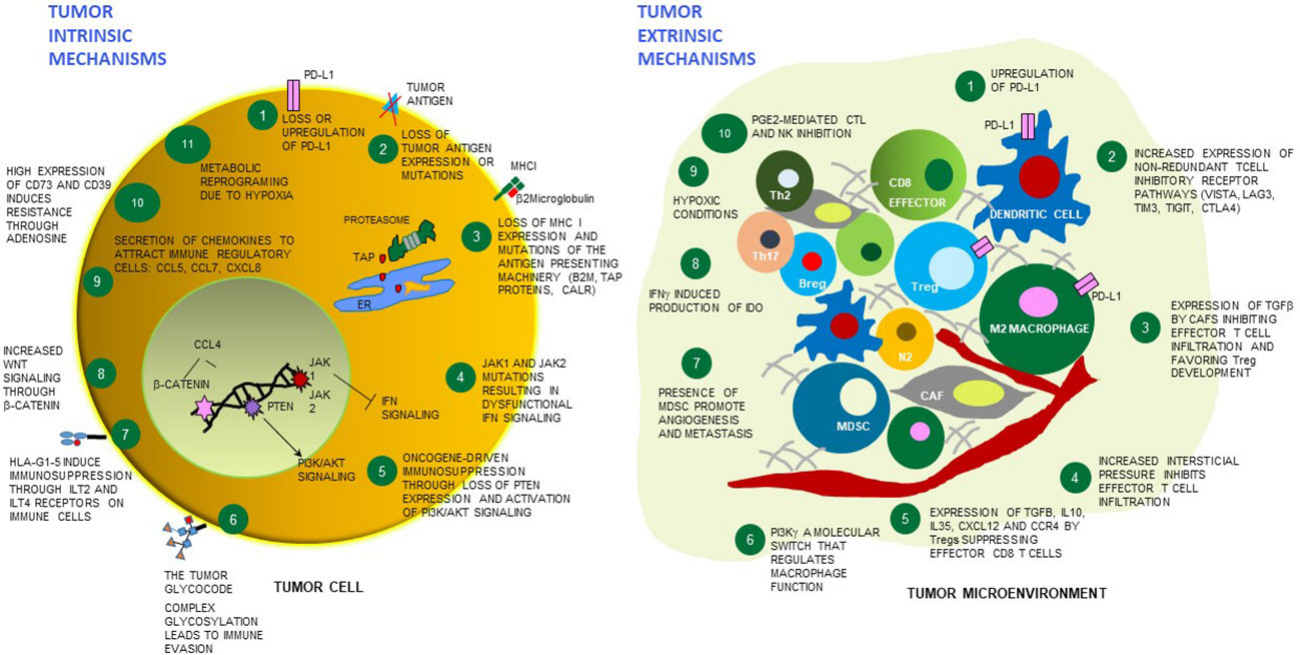
Tumor cell intrinsic and extrinsic mechanisms of resistance to immunotherapies. Schematic cartoon of some mechanisms of resistance to immunotherapies highlighting 11 tumor cell-dependent relevant mechanisms (left), and 10 mechanisms dependent on the microenvironment surrounding the tumor (right). Among the tumor extrinsic mechanisms, distinct immune cell types and stroma/endothelial cells are depicted which play a contributing role - or are affected by the overall TME immunosuppression. PD-L1 - programmed death-ligand 1; MHC-I - major histocompatibility complex class I; TAP - Transporter associated with antigen processing protein complex; ER, endoplasmic reticulum; CALR - calreticulin; JAK1 - Janus kinase 1; JAK2 - Janus kinase 2; B2M - beta-2 microglobulin; IFN - interferon; PTEN- phosphatase and tensin homolog protein; PI3K/AKT - phosphoinositide 3-kinase/Protein kinase B; HLA-G - human leukocyte antigen G; ILT2- Human inhibitory receptors Ig-like transcript 2; ILT4 - Human inhibitory receptors Ig-like transcript 4; CCL5 - C-X-C motif chemokine ligand 5; CCL7 - C-X-C motif chemokine ligand 7; CXCL8 - C-X-C motif chemokine ligand 8; CCL4 - C-C motif chemokine ligand 2; VISTA - V-domain Ig suppressor of T cell activation; LAG3 - lymphocyte-activation gene 3, TIM3 - T cell immunoglobulin and mucin domain-containing 3; TIGIT - T cell immunoreceptor with Ig and ITIM domains; CTLA4 – cytotoxic T-lymphocyte associated protein 4; TGF-beta - transforming growth factor beta; Tregs – regulatory T cells; IL-10 – interleukin10; IL-35 – interleukin 35; CXCL12 - C-X-C motif chemokine ligand 12; CCR4 - C-C chemokine receptor 4; PI3K gamma - phosphoinositide 3-kinase gamma; MDSC, myeloid-derived suppressor cells; IDO1 - indoleamine 2,3-dioxygenase enzyme, PGE2 - prostaglandin E2; CAF – cancer associated fibroblasts; NK – natural killer cells; N2 – neutrophil type 2; M2 macrophage – macrophages type 2; Breg – B regulatory cells; Th17 – T helper 17 cells; Th2 – T helper 2 cells.

Inflamed tumors are characterized by dense infiltration of CTLs, increased interferon gamma (IFN-gamma) signaling, expression of immune checkpoint markers (including PD-L1) (5), and high TMB. Tumors with an excluded T cell phenotype are characterized by the presence of T cells in the desmoplastic stroma surrounding the tumor. Despite these cells being recruited to the TME, there are obstacles hindering their infiltration into the tumor. The barriers can be the result of high levels of transforming growth factor beta (TGF-beta) (6), high hyaluronic acid levels (7), and/or the presence of abnormal desmosomal proteins (8) (**Figure 1**). Other factors limiting CTLs infiltration comprise cytokine and chemokine gradients, vascular endothelial growth factor (VEGF)-mediated immune suppression as well as numerous tumor-associated immune and stromal suppressive mechanisms (9) (**Figure 1**). The effects of TGF-beta, produced by tumor, stromal and immune cells within the TME, include promoting cancer-associated fibroblasts (CAFs) differentiation, induction of chronic tumor fibrosis and fibroblast to myofibroblast transition; moreover, TGF-beta facilitates the development of T regulatory cells (Tregs) and participates in extracellular matrix (ECM) remodeling (10) (**Figure 1**). Immunologically naïve (non-inflamed, cold) tumors tend to be genomically stable, contain fewer number of CTLs and are characterized by rapidly proliferating tumor cells (4).

Some authors have added a fourth category to this classification, “overheated” TME, to describe excessive inflammation that could impair the cytolytic activities of CTLs, triggering immune escape. This intense inflammation can be mediated by antitumor factors such as type 1 IFN, which are able to stimulate the expression of T cell inhibitory molecules on tumor cells, driving adaptive resistance to immunotherapy (11).

There are several theories describing tumor differentiation and growth. In the Darwinian clonal model, all cancer cell subclones possess tumorigenic potential, whereas in other models only a small subgroup of cancer cells, known as cancer stem cells (CSCs), can generate new tumors (12). In the latter model, CSCs can indefinitely self-renew or differentiate into multiple cancer cell types. CSCs could be more drug-resistant than other cancer cells and could be responsible for cancer recurrence and drug evasion (13). Increasing evidence suggests that various cancer cells can convert to a CSC state due to cell plasticity, e.g., due to epithelial-to-mesenchymal transition (EMT). Different subsets of CSCs with variable EMT phenotypes can coexist in tumors and switch from one to another (14). CSCs stemness and plasticity may be modulated by genetic, epigenetic and TME factors (15). Stem cell features could be acquired by cancer cells through clonal selection, however, we would like to highlight that the clonal evolution and the CSCs theories may not be mutually exclusive (16). Emerging data suggest that tumors may follow different models of evolution sequentially or simultaneously during the disease (17), but the full context of tumor evolution is still to be explored (18).

## 2 Oncogenic mechanisms leading to immune evasion and possibilities of therapeutic approach

The mechanisms of tumor cell escape may be classified into three main categories (19), namely reduced immune recognition, resistance mechanisms against CTLs, and genomic alterations in tumor-expressed tyrosine kinase pathways.

### 2.1 Reduced immune recognition

Reduced immune recognition includes loss of tumor antigens, antigen presenting cells or lack of costimulatory molecules. In this category decreased major histocompatibility complex class I (MHC-I) expression on tumor cells (**Figure 1**), decreased priming and activation of T cells and dendritic cells (DC), decreased expression of tumor-associated antigens (TAAs) and tumor-specific mutant antigens (TSMAs) can be observed. immune recognition could be a result of decreased MHC-I expression on tumor cells, decreased priming and activation of T and dendritic cells within the TME, or decreased expression of TAAs and TSMAs on tumor cells. This list is not comprehensive, and newer mechanisms are being added by current studies; moreover, these mechanisms could co-exist at a given time.

#### 2.1.1 Decreased MHC-I expression on tumor cells

**Figure 2:**
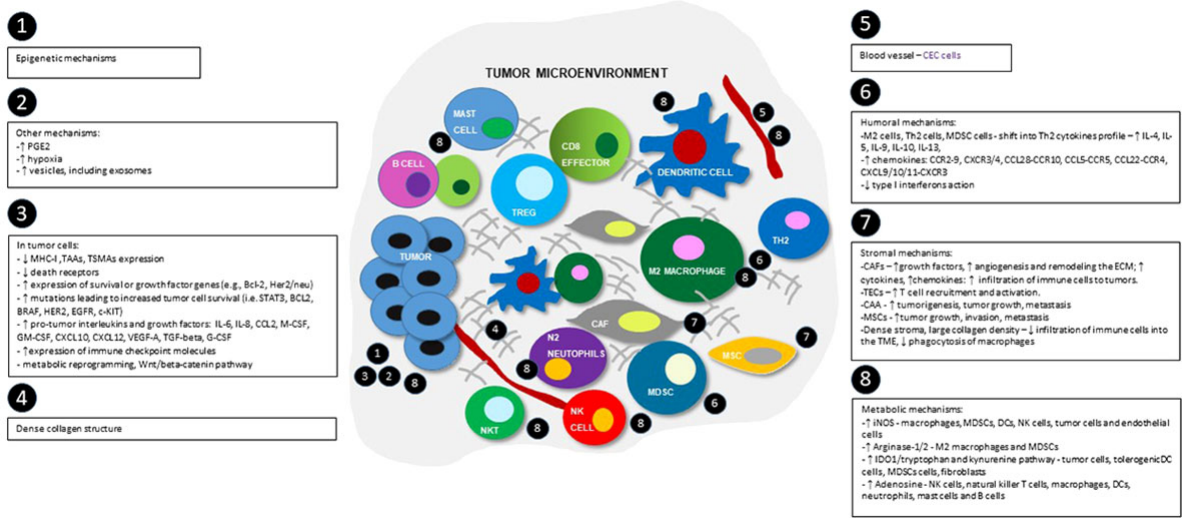
Schematic view of tumor microenvironment and the most important immunosuppression mechanisms, divided into epigenetic, tumor-cells dependent, humoral, metabolic, stromal and others groups. Numbers show the main locations of the processes within TME. MHC-I - major histocompatibility complex class I; TAAs- tumor-associated antigens; TSMAs - and tumor-specific mutant antigens; BCL-2 - B-cell lymphoma 2 protein; HER2 - human epidermal growth factor receptor 2; STAT3 - signal transducer and activator of transcription 3 pathway; BRAF - B-RAF proto-oncogene serine/threonine kinase; EGFR - epidermal growth factor receptor; c-KIT - tyrosine-protein kinase; IL-4 – interleukin 4; IL-5 - interleukin 5; IL-6 – interleukin 6; IL-9 – interleukin 9; IL-8 – interleukin 8; IL-10 - interleukin 10; IL-13 - interleukin 13; CCL2 - C-C motif chemokine ligand 2; M-CSF - macrophage colony stimulating factor; GM-CSF - granulocyte-macrophage colony-stimulating factor; G-CSF – granulocyte colony-stimulating factor; CXCL10 - C-X-C motif chemokine ligand 10; CXCL12 - C-X-C motif chemokine 12; VEGF-A - vascular endothelial growth factor A, TGF-beta - transforming growth factor beta; M2 cells - M2 phenotype macrophages; Th2 cells - Th2 helper; MDSC cells - myeloid-derived suppressor cells; CAFs – cancer associated fibroblasts; TECs – tumor associated endothelial cells; CAAs – cancer associated adipocytes; CCR2-9 - CC-chemokine receptor 2-9; CXCR3/4 - C-X-C chemokine receptors 3 and 4; CCL28-CCR10 - chemokine C-C motif ligand 28/C-C chemokine receptor type 10; CCL5-CCR5 - C-C chemokine ligand 5/C-C chemokine receptor type 5; CCL22-CCR4 - C-C motif chemokine ligand 22/C-C chemokine receptor 4; CXCL9/10/11-CXCR3 - CXC motif chemokine ligand 9/10/11 - C-X-C motif chemokine receptor 3; ECM – extracellular matrix; iNOS - inducible nitric oxide synthase; IDO1 - indoleamine 2,3-dioxygenase enzyme.

Tumors can avoid tumor-associated antigen presentation and T cell-mediated cytotoxicity via downregulation (20) or irreversible loss of MHC class I expression (**Figures 1**, **2**). HLA class I molecules are heterodimers consisting of heavy and light (beta-2 microglobulin) chains. Alterations of the HLA class I phenotype can result from mutations or deletions in genes encoding the HLA class I heavy chains on chromosome 6p21 or the beta-2 microglobulin gene encoding the light chain located on chromosome 15q21 (21). This may result in irreversible loss of heterozygosity. It was found that loss of one copy of an MHC-I heavy chain gene decreases MHC-I expression by 50% (22). In such cancer cells an inactivating mutation in the remaining MHC-I gene leads to a null phenotype (23). This phenotype could impair the defense against tumors by CTLs, but also, it could decrease the efficacy of immunotherapies restoring cytotoxic CTLs activity [e.g., checkpoint blockade (24) adoptive cell immunotherapy (25)]. When MHC-I is lost or downregulated, the absence of inhibitory MHC-I signals leads to an increased host response and enhanced natural killer (NK) cell cytotoxicity (26). However, cancer cells hijack this mechanism by producing factors such as TGF-beta and PGE2, impairing NK-cell function and blocking their infiltration into the tumor (27). Again, malignant cells may temporarily increase MHC-I expression, so they can avoid recognition by NK and T cell-mediated cytotoxicity (28). Altogether, impaired MHC-I antigen processing and presentation was found to be a predictor of acquired resistance to checkpoint inhibitors (CPI) therapy (29) and adoptive cell therapy (30). Potential therapeutic strategies to overcome this mechanism include inducing MHC-I expression in cancer via nuclear factor kappa beta (NFκB) stabilization, regulation of NFκB expression, or inducing MHC-I expression via restored IFN signaling. At the beginning of 2023 there were 85 recruiting and not yet recruiting clinical trials assessing different combinations of immunotherapy with no or low MHC antigen expression in different indications (31).

#### 2.1.2 Decreased priming and activation of T and dendritic cells impairs cytotoxic activity within the TME

Cytokines and growth factors present in the TME e.g., IL-6, IL-10, M-CSF, VEGF and TGF-beta were found to negatively regulate DC functions (32), inhibit DC differentiation from progenitors, and promote DCs differentiation into immunosuppressive cells such as MDSCs and tumor associated macrophages (TAMs) (33). Additionally, matrix metalloproteinase 2 (MMP-2) can change DC cells function to induce immunosuppressive Th2 (T helper 2 cells) responses (34). Signaling pathways such as beta-catenin, mitogen-activated protein kinases **(**MAPK) and signal transducer and activator of transcription molecules (STATs) play critical roles in the crosstalk between tumor cells and DCs in the TME. For example, increased beta-catenin signaling was shown to inhibit the recruitment of T cells and DCs into tumors (35). Moreover, melanoma-derived Wnt ligand (Wnt5alpha) was found to increase the production of IDO1 by DCs, leading to increased generation of Treg cells (36) (**Figure 1**).

#### 2.1.3 Decreased expression of TAAs and TSMAs by tumor cells

TAAs or TSMAs can be recognized by T cells, causing tumor cell death or selection of tumor escape clones. Decreased expression of TAAs and TSMAs enables faster tumor growth and inhibits tumor cell destruction (37) (**Figure 1**). Tumor antigen-specific T cells are present in progressively growing tumors, but they often present an exhausted state. These cells can be reactivated following treatment with anti-PD-1- and anti-CTLA-4 antibodies. TSMA and TAAs can be utilized for the development of personalized cancer-specific vaccines. Therapeutic approaches to overcome this immune escape mechanism include induction of immunogenic cell death (radiotherapy) adoptive cellular transfer therapy [e.g., chimeric antigen receptor T cells (CAR-Ts)] or adjuvants (CD40, CD137 and OX-40 agonists) as single agents and in combination therapies.

### 2.2 Resistance mechanisms against cytotoxic cells

Resistance mechanisms against CTLs, as well as increased expression of survival proteins [e.g., B-cell lymphoma 2 protein (BCL-2)] and tyrosine kinase receptors overexpression [e.g., human epidermal growth factor receptor 2 (HER2/neu)] can be developed by cancer cells (**Figure 2**). This leads to the survival of resistant tumor cells and an increase in the number of mutations [e.g., in HER2, BCL-2, signal transducer and activator of transcription 3 pathway (STAT3), B-RAF proto-oncogene, serine/threonine kinase (BRAF), epidermal growth factor receptor (EGFR), tyrosine-protein kinase KIT (KIT) genes]; applicable therapeutic strategies include targeting oncogenes and tyrosine kinase receptors e.g., BRAF inhibitors or anti-HER2 antibodies, small molecules, antibody-drug conjugates, and vaccines. Inhibiting cytotoxic cells could be mediated via inhibiting death receptor-mediated cytotoxicity, inhibiting granule-related cytotoxicity, tumor necrosis factor alpha (TNF-alpha) mediated cytotoxicity or via inhibiting the apoptotic pathway

#### 2.2.1 Inhibiting death receptor-mediated cytotoxicity: Fas ligand and tumor necrosis factor-related apoptosis inducing ligand

Cytotoxic lymphocytes may destroy target cells via the expression of death receptor ligands such as Fas and TRAIL. These ligands are transmembrane proteins expressed on cytotoxic immune cells (38). Both ligands trigger proapoptotic signaling. FasL binds to the Fas receptor and TRAIL binds to the death receptors 4 and 5 (DR4/5) (39). After binding of FasL or TRAIL, the death-inducing signaling complex (DISC) is created. DISC stimulates signaling leading to the activation of the mitochondrial apoptosis pathway, similar to granzyme B. This signaling can be inhibited by the activity of FADD-like IL-1 beta converting enzyme (FLICE)-inhibitory proteins (FLIPs), expression of decoy receptors, or downregulation of death receptors by tumor cells.

#### 2.2.2 Inhibiting TNF-mediated cytotoxicity

TNF-alpha is a cytokine capable of inducing both pro-survival and pro-apoptotic signaling. The receptors for TNF-alpha, tumor necrosis factor receptor 1 (TNF-R1) and tumor necrosis factor receptor 2 (TNF-R2) belong to the same family as FasL and TRAIL receptors, but the downstream signaling pathways are different. TNF-R1 receptor is able to trigger cell death via the cytoplasmic death domain, which recruits a TNF receptor-associated death domain (TRADD) (40). On the contrary, both TNF-R1 and TNF-R2 contain a TNFR-associated factor (TRAF) binding site that recruits TRAF1/2, involved in triggering pro-survival signaling via the NFκB and MAPK pathways. As both TNFR receptors are highly expressed on Tregs, targeting TNFR was considered a promising immunotherapeutic approach. Therefore, TNFR2 antagonists can block both immunosuppressive cells and tumor cells.

#### 2.2.3 Inhibiting granule-mediated cytotoxicity

Perforins and granzymes are secreted by cytotoxic T cells and NK cells (41). Resistance mechanisms exploited by cancer cells include reluctance to perforin pore formation in target cells (reduced cell stiffness to prevent efficient perforin pore formation), changes in the cell membrane lipid order in tumor cells (42), changes in the glycosylation patterns of protein components in the cancer cell membrane (glycocode) (**Figure 1**), as well as secretion of cathepsin B to degrade perforins. Other observed mechanisms of resistance to granzyme-mediated apoptosis encompass resistance to autophagy and to gasdermin-induced pyroptosis (43).

#### 2.2.4 Inhibiting apoptotic pathways

Prevention of cancer cell apoptosis may occur via downregulation of pro-apoptotic mediators, including caspases or pro-apoptotic BCL-2 family members, or up-regulation of apoptosis inhibitors, such as inhibitor of apoptosis proteins (IAPs) or anti-apoptotic BCL-2 family members. The *BCL-2* gene encodes a family of proteins critical for apoptosis regulation. This family includes proteins promoting cell survival e.g., BCL-2 and B-cell lymphoma-extra large (BCL-x_L_); initiating cell death e.g., BCL-2-interacting mediator of cell death (BIM), p53 upregulated mediator of apoptosis (PUMA), BCL-2-interacting domain (BID); or activating the effector pathways of apoptosis (BAK) (44).

Therapeutic approaches inhibiting NK and CTL activity are being extensively studied in cancer. There were 150 trials investigating the TRAIL pathway as of the beginning of 2023 (31). BCL-2 inhibitors, playing an especially important role in hematological malignancies, are also intensively studied, which is reflected in a high number of studies aiming to inhibit this pathway either in combination with other immunotherapies or with chemotherapeutic agents.

### 2.3 Genomic alterations in tumor-expressed tyrosine kinase pathways

A pro-tumor microenvironment can be established via the secretion of pro-tumor cytokines, chemokines and growth factors such as interleukin 4 (IL-4), interleukin 6 (IL-6), interleukin 8 (IL-8), interleukin 10 (IL-10), C-C motif chemokine ligand 2 (CCL2) (45); C-C motif chemokine 22 *(*CCL22); C-C motif chemokine ligand 24 (CCL24); macrophage colony stimulating factor (M-CSF), TGF-beta, C-X-C motif chemokine ligand 10 (CXCL10), C-X-C motif chemokine 12 (CXCL12), VEGF-A and granulocyte colony-stimulating factor (G-CSF), (46), as well as metabolic factors including adenosine, prostaglandin E2 (PGE2), and indoleamine 2,3-dioxygenase (IDO1) (47) (**Figure 2**). These factors stimulate the recruitment of regulatory T cells, myeloid-derived suppressor cells (MDSCs) and regulatory B cells (Bregs) and enhance adaptive immune resistance via an increase in the expression of inhibitory receptors on CTLs e.g., cytotoxic T-lymphocyte antigen 4 (CTLA-4); programmed cell death protein 1 (PD-1), T cell immunoglobulin and mucin domain-containing 3 (TIM-3) (**Figure 1**). Possible therapeutic strategies include checkpoint blockade, e.g., blockade of CTLA-4, PD1, PD-L1; targeting angiogenesis, e.g., anti-VEGF drugs; or inhibiting tumor-specific metabolic pathways, e.g., IDO1 inhibitors. A short description of each category is presented below and graphically on **Figure 1** and **Figure 2**, with the examples of the current therapies in clinical trials shown in **Table 1**.

**Table 1:** Examples of current clinical trials targeting TME mechanisms.

Mechanism of action	Name of drug	target	Phase of trial	Type of cancer	Concomitant therapies	NCB identifier/reference
inducing MHC-I expression - NF-kappa beta stabilization, regulation of NF-kappa beta expression restored IFN signaling	Sodium stibogluconate	SHP2	2	Myelodysplastic Syndromes/Acute Myeloid Leukemia With One of the 65 Defined p53 Mutations	NA	NCT04906031
WNT/beta pathway modulators	DKN-01	Dickkopf-1 (DKK1) is a modulator of the Wnt and PI3K/AKT signaling pathways	1, 2	patients with advanced mismatch repair proficient oesophagogastric cancer	Atezolizumab	NCT04166721
STING agonists	E7766 (eisai)	highly potent STING agonist	1/1b	patients with advanced solid tumors or lymphomas	NA	NCT04144140
Inhibiting Death Receptor-Mediated Cytotoxicity	IGM-8444	DR5 (death receptor 5)	1	patients with relapsed and/or refractory solid cancers	FOLFIRI Chemotherapy Regimen	NCT04553692
Inhibiting Granule-Mediated Cytotoxicity	Pre-clinical studies	NA	NA	NA	NA	NA
Inhibiting Apoptotic Pathways Bcl-2 inhibitors	BGB-11417	highly selective Bcl-2 inhibitor	1	patients with mature B-cell malignancies	NA	NCT04883957
mutations leading to increased tumor survival - HER2 pathway	pyrotinib	pan-HER2 inhibitor	2	HER2-positive Advanced Colorectal Cancer patients	trastuzumab	NCT04380012
mutations leading to increased tumor survival - BRAF mutations	HLX208	BRAF V600E Inhibitor	2	metastatic colorectal cancer patients	NA	NCT05127759
mutations leading to increased tumor survival - EGFR pathway	DZD9008	selective, irreversible EGFR inhibito	1/2	patients with advanced non-small cell lung cancer with EGFR or HER2 mutation	NA	NCT03974022
mutations leading to increased tumor survival - c-KIT pathway	Imatinib	BCR-ABL, c-KIT, PDGFRA	2	patients with stage III unresectable and stage IV melanoma with C-KIT gene mutation	Toripalimab	NCT05274438
targeting MDSC cells - phosphatidylinositol 3-kinase (PI3K) delta	IPI-549	specific PI3K gamma inhibitor	2	Patients With Locally Advanced HPV+ and HPV- Head and Neck Squamous Cell Carcinoma	NA	NCT03795610
Adoptive cell therapies: CAR-T	CEA CAR-T cells	CAE + cancer cells	1,2	patients with relapsed and refractory CEA+ cancer	NA	NCT04348643
Adoptive cell therapies: CAR-NK	NKG2D CAR-NK	NKG2D	1	patients with refractory metastatic colorectal cancer	NA	NCT05213195
Adoptive cell therapies: CAR-P	No studies – pre-clinical	NA	NA	NA	NA	NA
Cytokine based therapies	NKTR-214 (Bempegaldesleukin)	CD122-preferential IL2 pathway agonist	1	mCRPC patients who received prior secondary androgen receptor signaling inhibitor therapy	Nivolumab, radiation, Stereotactic body radiation therapy (SBRT), CDX-301, Poly-ICLC, INO-5151,	NCT03835533
Chemokine based therapies	Rintatolimod	selective TLR3 agonist, immune response modulator	1	patients with early stage triple negative breast cancer	Drug: CyclophosphamideDrug: DoxorubicinDrug: Doxorubicin HydrochlorideDrug: PaclitaxelBiological: Recombinant Interferon Alfa-2b	NCT04081389
inhibiting NO/iNOS pathway	Pre-clinical	NA	NA	NA	NA	NA
Inhibition of ARG1/2 activity	Arginase1 peptide: ArgLong2 (169-206) Peptide sequence	Arg-1 inhibitor	1,2	patients with polycythemia vera, essential thrombocythemia	Vaccine - PD-L1 peptide: PD-L1 Long (19-27)	NCT04051307
blocking of adenosine pathway - targeting A2A adenosine receptors	ILB2109	A2AR antagonist	1	patients with advanced solid malignancies	NA	NCT05278546
targeting the IDO pathway	Epacadostat	inhibitor of indoleamine 2,3-dioxygenase-1 (IDO1)	1,2	patients with metastatic or locally recurrent breast cancer patients	Retifanlimab, SV-BR-1-GM, low dose cyclophosphamide Interferon Inoculation	NCT03328026
blockade of PGE2 pathway	CR6086	selective small molecule antagonist of the prostaglandin E2 receptor, EP4 subtype	1, 2	patients with pretreated mismatch-repair-proficient and microsatellite stable metastatic colorectal cancer	Balstilimab	NCT05205330
Hypoxia - targeting HIF pathway	DFF332	HIF-2 blockade	1	patients with advanced/relapsed ccRCC and other malignancies with HIF2α stabilizing mutations	RAD001, PDR001, NIR178	NCT04895748
exosomes	Mesenchymal Stromal Cells-derived Exosomes with KRAS G12D siRNA	KRAS mutation	1	patients with metastatic pancreas cancer with KrasG12D mutation	NA	NCT03608631
Antibodies bi- and tri-specific combinations	FS118	PD-L1, LAG-3	1, 2	patients with advanced malignancies	NA	NCT03440437
Anti-cancer vaccines	ACIT-1	Stimulation of tumour antigen-specific T cells to respond and kill cancer cells	1, 2	patients with pancreatic and other cancers	NA	NCT03096093
radiation	radiation therapy	numerous targets	2	patients with unresectable non-metastatic pancreatic cancer	Gemcitabine, Capecitabine	NCT01972919
nanoparticles	WGI-0301	Lipid Nanoparticle Suspension of Akt-1 Antisense Oligonucleotide	1	patients with advanced solid tumors	NA	NCT05267899
Immune checkpoint molecules - TIM-3 inhibitors	Cobolimab	anti-TIM-3	2	melanoma stage IV patients	TSR-042	NCT04139902
Immune checkpoint molecules – LAG-3 inhibitors	INCAGN02385	Anti-LAG-3	1, 2	patients with selected advanced malignancies	INCAGN02390 INCMGA00012.	NCT04370704
Immune checkpoint molecules – TIGIT inhibitors	COM902	TIGIT Inhibitor	1	subjects with advanced malignancies	COM701	NCT04354246
Immune checkpoint molecules - CTLA-4,	Tremelimumab	human monoclonal antibody against CTLA-4	1, 2	patients with gastroesophageal cancer and other gastrointestinalmalignancies	Cabozantinib, Durvalumab	NCT03539822
Immune checkpoint molecules - PD-1	GLS-010 (zimberelimab)	Fully Human Anti-PD-1 Therapeutic Monoclonal Antibody	2	patients with recurrent or metastatic cervical cancer	NA	NCT03972722
Epigenetic mechanisms - HDAC Inhibitors (HDACi)	Chidamide	selective histone deacetylase (HDAC) inhibitor	1, 2	patients with advanced cervical cancer	Toripalimab	NCT04651127
Epigenetic mechanisms - Histone Methyltransferase Inhibitors (HMTi/EZH2i	tazemetostat	EZH2 inhibitor or - histone methyltransferase inhibitor	2	subjects with relapsed/refractory follicular lymphoma	rituximab	NCT04762160
Epigenetic mechanisms - Histone Reader Protein Inhibitors (bromodomain and extra-terminal domain proteins - BETi)	ZEN-3694	BET bromodomain inhibitor	2	patients with metastatic castration resistant prostate cancer	enzalutamide; pembrolizumab	NCT04471974
Epigenetic mechanisms - DNA methyltransferase inhibitors	Azacitidine, decitabine	demethylation agents	2	patients with newly diagnosed acute myeloid leukemia	Several concomitant drugs	NCT03164057
Stromal mechanisms - CAFs targeted therapies	Pritumumab	ecto-domain of vimentin on the surface of cancer cells	1	patients with brain cancer	NA	NCT04396717

The analysis of human tumors has shown that tumorigenesis can be driven by gain of function mutations in cell death antagonists or loss of function mutations in cell death activators. These mutations can serve as initiating events or as secondary oncogenic events to promote tumor development and progression to metastatic disease (48). Oncogenic mutations causing deregulated activation of receptor tyrosine kinases or their downstream signaling pathways are frequent in human cancers (49). As an example, presence of *HER2* gene amplification was found in 10%–34% of invasive breast cancers (50). Among other crucial alterations, *RAS (KRAS, NRAS, HRAS)* gene mutations are found in approximately 27% of all cancers, with high frequency of *KRAS* mutations in pancreatic duct adenocarcinoma, lung adenocarcinoma. Missense gain-of-function mutations in the *RAS* genes occur with 98% of the mutations at one of three mutational hotspots: G12, G13 and Q61 (COSMIC v75). Mutant *RAS* is considered to negatively impact GTP hydrolysis, which results in an accumulation of constitutively GTP-bound RAS proteins in cells (51). Another important pathway is associated with activating mutations in the phosphatidylinositol-4,5-bisphosphate 3-kinase catalytic subunit alpha (*PIK3CA*) gene occurring in approximately 30–40% of patients with cancer (52). Analysis of this gene in human tumor samples identified hotspot mutations in three sites, E542 and E545 in the helical domain (exon 9) and H1047 in the kinase domain (exon 20) (53), which induce activation of the alpha isoform of *PI3K*. Another gene contributing to increased cancer cell survival is *BRAF*, encoding a serine/threonine kinase protein and engaged MAPK pathway. Somatic mutations of *BRAF* gene are found in up to 15% of human tumors (54), including melanomas, and papillary thyroid carcinomas (55). The most common *BRAF* mutation is the V600E change in exon 15 which activates the BRAF kinase activity via phosphorylation (56). The protein encoded by the *KIT* gene, c-KIT, is a stem cell growth factor receptor, one of the type III receptor tyrosine kinases known to play a critical role in the onset and proliferation of cancer. Activating mutations in *KIT* are considered the molecular drivers of gastrointestinal stromal tumors. Most *KIT* mutations happen in exon 11, and the deletions are most commonly found in codons 557 and 558 (57). Among other pathways engaged in increased survival of cancer cells there are *STAT3* family of genes regulating cellular proliferation, apoptosis and angiogenesis. Mutations leading to the constitutive activation of *STAT3*, are important in oncogenesis in both solid and hematological malignancies (58). Further pathway leading to increased cancer cell survival are mutations associated to *EGFR* overexpression, found in adenocarcinoma of the lung (59), and colorectal cancer (60). Although these findings are not a complete list, they confirm the view that, deregulated mitogenic signaling is a major driver of cancer development (61).

Therapeutic approaches to overcome increased tumor cell survival include the combination of histone deacetylases (HDAC) and MAPK inhibition, selective BRAF inhibitors (e.g., vemurafenib or dabrafenib), MEK1/2 pathway inhibition (trametinib) or a BRAF/MEK inhibitors combination in patients with confirmed mutations (62). Drugs targeting KRAS are being developed, with the example of KRAS (G12C) inhibitors, which led to FDA approval of sotorasib in 2021and adagrasib in 2022 (63) for patients with KRAS-mutated non-small cell lung cancer (NSCLC) (64). Other strategies are also assessed in clinical trials, including antibody-drug conjugates [e.g., ado-trastuzumab emtansine (T-DM1)] designed to target HER2 and release a cytotoxic drug in NSCLC patients with *HER2* mutations. The antibody-drug conjugate, 4C9-DM1, is an example of a c-KIT targeting drug in development (65).

First generation EGFR tyrosine kinase inhibitors (EGFR-TKIs) such as gefitinib and erlotinib or second-generation molecules such as afatinib and dacomitinib are effective for the treatment of *EGFR*-mutated NSCLC, mainly in patients with *EGFR* exon 19 deletions or an exon 21 L858R mutation. A compounding issue is that a majority of patients face a cancer recurrence within 2 years due to acquired therapy resistance, mostly associated with the *EGFR* T790M mutation in exon 20. A third generation TKI, e.g., osimertinib targeting the T790M mutation, was developed to overcome such resistance and showed high clinical efficacy. However, resistance to third generation TKIs was observed through a C797S mutation (66). Current therapeutic options address patients with the so called triple mutation: T790M, L858R or exon19 deletion, and C797S mutation (fourth generation EGFR inhibitors), as well as patients with non-resistant rare EGFR mutations, including L861Q, G719X and S768I.

Both small molecule inhibitors and targeted antibodies used in cancer immunotherapy have their advantages. Small molecule inhibitors usually bind a wider number of targets in comparison to antibodies due to their smaller size. Antibodies are more specific but they are characterized by poor tumor penetration and immunogenic potential (67). However, small molecule inhibitors and therapeutic antibodies can be considered complementary strategies in cancer treatment, and can be combined to achieve synergistic effects. Examples are monoclonal antibodies targeting EGFR. These antibodies block the extracellular ligand binding domain of the receptor and signal molecules cannot longer activate the tyrosine kinase. These therapeutics include cetuximab, panitumumab, nimotuzumab, zalutumumab, or duligotuzumab, the novel humanized dual EGFR/HER3 monoclonal antibody. Drugs targeting mutations in receptor tyrosine kinases, as well as in their downstream signaling members, are one the most actively developed anti-tumor drugs. This is reflected in the high number of trials ongoing at the beginning of 2023, with 1004 studies targeting the HER2 pathway, 246 trials targeting *BRAF* mutations, 773 studies targeting the EGFR pathway and 291 studies targeting the c-KIT pathway (31).

The mutations leading to increased cancer cell survival, discussed earlier, are engaged in the activation of oncogenic pathways, including WNT/beta-catenin, STAT3, PI3K/PTEN/AKT/mTOR, RAS/RAF/MAPK or NFκB (68). These signaling pathways can influence exclusion and dysfunction of cytotoxic cells in the TME (69) (**Figure 1**). There were 13 trials assessing different combinations of immunotherapy with novel WNT pathway modulators, including DKN-01or LGK974 in various forms of cancers at the beginning of 2023 (31).

### 2.4 The immunosuppressive TME

The mutual interactions of immune and non-immune cells, humoral, metabolic and other factors present in the TME lead to an immunosuppressive environment outlined below.

#### 2.4.1 Immune-cell dependent mechanisms

Several myeloid and lymphoid cell types in the TME play important roles in immune suppression mechanisms (70). M2 macrophages are abundant (up to 50% of tumor mass) (71) and they are probably the most important population of immunosuppressive cells in the TME secreting scavenger receptors, pro-tumorigenic cytokines, chemokines and pro-angiogenic factors (72). Another cell population, MDSCs, contribute to tumor growth via regulating the adenosine metabolism, expression of negative immune checkpoint molecules, and a shift towards the immunosuppressive Th2 response. Pro-tumor N2 neutrophils are known to influence cancer progression by the secretion of C-X-C motif chemokine ligand 1 (CXCL1), matrix metallopeptidase 9 (MMP-9), VEGF, and TNF-alpha (73), as well as ROS and NO (74). Another crucial population of lymphoid cells, Tregs, mainly inhibit tumor-specific T cell responses. Th17 cells are characterized by the secretion of immunosuppressive interleukin 17F (IL-17F), interleukin 17A (IL-17A), IL-6, interleukin 21 (IL-21), interleukin 22 (IL-22), and IL-23 (75). Th2 cells contribute to immune tolerance mainly by the secretion of protumor Th2 cytokines. The protumor action of Bregs involve secretion of IL-10 (76) as well as production of granzyme B and TGF-beta (77). The immune cells’ phenotype shows plasticity and may depend on TME polarization into pro- or antitumor immunity (i.e. already differentiated M2 macrophages and N2 neutrophils cells could change their phenotype into antitumor M1 or N1 respectively) (78) (**Figure 1**, **2**). Various therapeutic approaches addressing the immune cell-dependent immunosuppression within the TME have been identified. These include among others including turning cold tumors into hot, targeting MDSCs, reprogramming of TAMs, or adoptive cell therapies.

##### 2.4.1.1 Turning immunologically cold tumors into hot

According to the previously described “cold” and “hot” TME phenotype, it seemed reasonable that “heating up” the TME, namely increasing immune infiltration in the TME, could enhance antitumor immunity. This therapeutic approach is not exclusively an immune-cell dependent mechanism, as humoral and metabolic factors can influence this process (79) (**Figure 1**). Several attempts have been implemented to turn cold tumors into hot, including the activation of the innate immune system using stimulators of interferon genes (STING) agonists, increasing cross-presentation of DCs to promote tumor-antigen-specific T cell infiltration into the TME, targeting the cellular metabolism or transferring within the TME certain metabolites to reduce Tregs, MDSCs or TAMs infiltration (80) as presented in **Figure 2**. An additional therapeutic approach has been to promote cytotoxic T cell activity or re-educate TAMs, MDSCs, and Tregs to support CTLs effector functions. Further attempts included creating an inflamed TME via oncolytic viruses or nanoparticle delivery of immune-modulatory factors (81).

##### 2.4.1.2 MDSCs targeting options

Blocking the immunosuppressive function of MDSCs could be achieved in several ways: by depleting MDSCs, through inhibition of their immunosuppressive potential, by decreasing MDSC cell recruitment to the tumor, or by modulating myelopoiesis. It has been shown that targeting phosphatidylinositol 3-kinase (PI3K) delta and PI3K gamma leads to the inhibition of NFκB and activation of CCAAT/enhancer-binding protein beta (CEBPbeta), which results in an inhibition of the MDSCs immunosuppressive activity (82) (**Figure 1**). Targeting both isoforms of PI3K in combination with a PD-L1 blocking antibody delayed tumor growth and prolonged survival in tumor models of head and neck cancer, indicating a beneficial effect of this treatment combination (83).

##### 2.4.1.3 Reprogramming/repolarization of TAMs

As mentioned previously, the immunosuppressive M2 TAMs can be repolarized into a M1 phenotype under certain circumstances. Another way to reprogram M2 TAMs is to use genetic engineering to enhance their antitumor activity (80). The TME immunosuppressive status was altered *in vitro* by genetically modified macrophages, which once transplanted into patients (84) enabled the stimulation of the cytotoxic activity of T cells *in vivo* and inhibited the immunosuppressive status of the TME (69).

##### 2.4.1.4 Advances in adoptive cell therapies

This treatment modality is a fast-developing field of cancer immunotherapy (85). Cells collected from a patient are genetically engineered, multiplied *ex-vivo* and infused back into the patient. The benefits of ACT could be enhanced by adding small-molecule drugs or epigenetic modulators to enhance T-cell expansion, and has been reviewed elsewhere in detail (86). The PI3K inhibitor idelalisib has been shown to inhibit human T reg functions (87). Inhibition of PI3K gamma and delta with duvelisib reprogramed differentiation and the metabolism of CAR-T cells, improving their expansion and anti-tumor cytotoxicity (88). Among tyrosine kinase inhibitors dasatinib showed promising activity of reversing T cell exhaustion, which translated into enhanced therapeutic efficacy (89).

Adding epigenetic modulators represent another strategy to improve T-cell function. DNA methyltransferases and histone deacetylases are activated during T-cell differentiation, resulting in high levels of DNA and histone methylation in exhausted T cells (90). It was shown, that decitabine, a DNA methylation inhibitor, enhances anti-tumor activities, cytokine production, and CAR-T cell proliferation in non-Hodgkin lymphoma models (91). Additionally, treatment of CAR-T cells with a BET inhibitor (92) or immune modulator drugs like lenalidomide, showed to enhance CAR T-cell response in hematological malignancies models (93).

Despite promising efficacy in hematological malignancies, the results of CAR-Ts in solid tumors remain unsatisfactory. Compared to hematological malignancies, solid tumors show higher tumor antigen heterogeneity, associated with effective escape mechanisms against mono-antigen-specific CAR-T cells. Another factor is a presence of the immunosuppressive TME demonstrating physical and molecular barriers preventing CTLs infiltration, driving their dysfunction and hypoproliferation (94).

Next-generation bi-specific CAR-Ts are being developed to overcome these challenges (95), these include fourth and fifth generation CAR-Ts delivering drugs able to modify the TME through the release of transgenic immune modulators (96). Chimeric antigen receptor macrophage-cells **(**CAR-M) can destroy tumor cells or alter the TME creating a niche of tumor and immune cells. Transduced human macrophages with an anti-HER2 CAR could be an example of such therapy. CAR-Ms were able to perform antigen-specific phagocytosis *in vitro*, leading to reduced tumors and prolonged overall survival in murine solid tumor models. An assessment of the effects of CAR-Ms on M2 macrophages found that CAR-Ms induced a phenotypic shift in M2 macrophages towards a M1 phenotype and activated cytotoxic T cells. As a result, CAR-Ms reprogrammed the TME, showing potential efficacy in solid tumors (97).

Alternative promising strategies are CAR-NKs based therapies. Compared to CAR-Ts, chimeric antigen receptor natural killers **(**CAR-NKs) have shown improved safety with few cases of cytokine release syndrome (CRS) and no graft versus host disease (GvHD) reported (98). In addition to their effectiveness in blood cancers, CAR-NKs are being investigated in solid tumors such as pancreatic, ovarian and prostate cancers. CAR-NKs therapies with their favorable cytotoxicity, short lifespan and lower manufacturing costs are considered the alternative to CAR-Ts (99).

##### 2.4.1.5 Clinical benefits of tumor infiltrating lymphocytes adoptive cell therapy

As CAR-T cell therapy has not yet shown convincing clinical benefit in the treatment of solid tumors, application of autologous TIL-ACT (tumor infiltrating lymphocytes adoptive cell therapy) is being explored as an alternate approach. TIL-ACT therapy starts with isolating the natural infiltrating lymphocytes from the tumor tissues, expanding them *in vitro*, and then infusing these cells back with a high dose of IL-2 to ensure anti-tumor efficacy. Prior to infusion of the TIL cells, patients receive a non-myeloablative lymphodepletion regimen. This therapy has shown efficacy in several indications including metastatic melanoma (100), cervical cancer (101), and breast cancer (102).

##### 2.4.1.6 Perspective of T cell receptor transduced T cell therapy

Another promising therapeutic alternative is therapy with T cells expressing an engineered T cell receptor (TCR-T cells). This approach could overcome a CAR-T cells limitation of targeting surface protein antigens only, frequently not expressed on solid tumors. In addition to surface antigens, TCR-T cells can target the intracellular antigens of solid tumors, ensuring enhanced anti-tumor efficacy (103). Autologous T-cell receptor (TCR)-based adoptive therapy is based on genetically modified lymphocytes against specific tumor markers. Ongoing clinical trials will determine the ultimate role of TCR-based therapies in patients with solid tumors (104). TCR-T cell therapy developed so far rely on engineering of autologous T cells. However, implementation of allogeneic TCR-T cell therapies could offer multiple advantages including immediate availability, standardization, and reduced cost compared to conventional CART cell therapies (105). By deleting both endogenous TCR alpha and TCR beta chains, insertion of the transgenic TCR would decrease the risk of graft-versus-host disease. This approach can be combined with strategies to limit the rejection of the allogeneic T cells by the host immune system, such as partial HLA matching or gene editing (HLA class I deletion combined with natural killer cell inhibition) to generate universal T cells (106). The first TCR-based therapy was recently approved by the US FDA (107).

Another related approach is the development of bispecific T cell engagers (BiTE) with a TCR component recognizing a tumor specific peptide antigen in the context of a particular HLA haplotype on one end, and a CD3 component to attract CTL effector cells to the tumor on the other end. BiTE therapeutics are small and flexible, easily diffusible to lesions, redirecting cytotoxic lymphocytes to cancer cells with high affinity (108). Monitoring HLA expression under these therapeutic treatments becomes a requirement, as tumors frequently evolve downregulating HLA expression as a mechanism of tumor immune evasion, limiting peptide antigen recognition by CTLs.

Adoptive cell therapies are gaining significant research attention reflected in the number of ongoing clinical studies. There are at least 197 TIL-ACT trials and 601 TCR-T cell trials ongoing. Moreover, there were 642 studies assessing CAR-Ts therapies in different combinations and 32 trials assessing CAR-NKs therapies at the beginning of 2023 (31).

Additional TME reprogramming possibilities include: the use of ligands for toll-like receptors (TLRs), such as the TLR7 agonist imiquimod; TLR9 agonists; CpG oligodeoxynucleotides or whole microorganism-based adjuvants, such as BCG (109).

##### 2.4.1.7 The role of immune checkpoint inhibitors

ICIs modulate innate or adaptive immune responses. They can be divided into two classes: ICIs that co-stimulate [TNF family members, CD27, 4-1BB (CD137), OX40 (CD134), herpesvirus entry mediator (HVEM), CD30, and glucocorticoid-induced TNFR-related protein (GITR)] (110) and ICIs that inhibit immune responses (111) such as PD-1, PD-L1, CTLA-4, VISTA, TIM-3, TIGIT, HLA-G and LAG-3 (**Figure 1**). ICIs form ligand-receptor pairs with other molecules, the receptors are mostly expressed on immune cells, while the ligands are mostly expressed on antigen-presenting cells, tumor cells, or other cell types, (112). Overexpression of these ligands on tumor cells can be the result of cell-autonomous stimuli or of stimuli from the TME. The activation of inhibitory ICIs causes the inhibition of cytotoxic T cells (113), NK and NKT cell functions. Exhausted cytotoxic T cells are unable to lyse tumor cells, they have impaired effector functions and they show an inability to product pro-inflammatory cytokines (e.g., TNF-alpha, IFN-gamma, IL-2). They express co-inhibitory receptors including CTLA-4, PD-1, TIM-3, TIGIT or LAG-3 (114). Ongoing research has helped discover novel checkpoint inhibitors such as B7-H3, B7-H4 transmembrane proteins, NKG2A proteins, PVRIG/PVRL2 (poliovirus receptor-related immunoglobulin domain), as well as inhibitory targets beyond immune checkpoints including carcinoembryonic antigen-related cell adhesion molecules 1, 5, 6 (CEACAM1, CEACAM5, CEACAM6), and focal adhesion kinase (FAK) (115).

###### 2.4.1.7.1 The role of TIM-3

TIM-3 is a transmembrane receptor expressed by CTLs, Tregs, B cells, macrophages, NK cells, DCs and tumor cells (116). The main ligands are galectin-9, phosphatidyl serine, and CEACAM1 (117). TIM-3 acts as an immune checkpoint promoting immune tolerance (**Figure 1**). Stimulation of TIM-3 by ligands causes T cell exhaustion and expansion of MDSCs within the TME. Finding that TIM-3 can be an immune checkpoint in the malignant TME came from the observation that TIM-3 was present on the suppressed CTLs in preclinical models of tumors, and the CD8 TIM-3+ T cells expressed also PD-1 (118). Moreover, TIM-3^+^ Tregs are rarely found in peripheral blood and lymphoid tissues. This indicates that TIM-3 can be specific to tissue Tregs and these cells could play more important role in suppressing anticancer immunity in tumor tissue (119). High TIM-3 levels correlated with poor prognosis in prostate, renal cell, colon, and cervical cancers. TIM-3 blockade results in decreased MDSCs and increased proliferation and cytokine production by T cells (120). Given its expression in a variety of T cells and its synergistic effects with other anti-PD-1 agents, TIM-3 blockade was assessed as an attractive therapeutic target, which was investigated in 43 clinical trials early in 2023 (31).

###### 2.4.1.7.2 The role of LAG-3 (CD223)

LAG-3 is another promising immune checkpoint therapeutic target together with PD-1 and CTLA-4. LAG3 interacts with MHC class II and it is expressed on CD4^+^ T cells, CD8 T cells, NK cells, NKT cells, Tregs (121), B cells and DCs (122). LAG3 has several ligands including MHC class II, lymph node sinusoidal endothelial cell C-type lectin, Galectin-3, alpha-synuclein, fibrinogen-like protein 1 and 2 (FGL1, FGL2) (123), all of which inhibit T cell activation through binding to LAG-3. LAG3 interaction with MHC class II causes a decrease of CTLs cytokine production, CD4 and CD8 T cell expansion, and supports a Treg phenotype differentiation to prevent tissue damage and autoimmunity (124) (**Figure 1**). Tumor-infiltrating lymphocytes can overexpress LAG-3, which contributes to their dysfunction and immune exhaustion (117). High LAG-3 and FGL1 expression has been shown to support tumor growth via accelerating T cell exhaustion and blocking T cell proliferation (125). LAG-3 has been also identified as a mechanism of resistance to some immunotherapies, including anti-PD-1 therapies. LAG-3 blockade stimulates immune activation against tumor cells and enhances the effect of other immune checkpoint inhibitors (126). In March 2022, relatlimab, the first monoclonal antibody targeting LAG-3 in combination with nivolumab, was approved by the FDA for the treatment of untreated/unresectable or metastatic melanoma. The RELATIVITY-047 (127) study demonstrated that this combination doubled the progression free survival (PFS) time compared to nivolumab alone. Other anti-LAG-3 agents are currently in development, including favezelimab, fianlimab, the bispecific tebotelimab, ieramilimab or INCAGN-2385. Numerous trials assessing LAG-3 across different cancer indications and in combinations could change the existing strategy for immunotherapies. There were 64 studies targeting LAG-3 in cancer patients at the beginning of 2023 (31).

###### 2.4.1.7.3 The role of TIGIT

T-cell immunoglobulin and ITIM domain (TIGIT) is expressed on dysfunctional T cells, Tregs and NK cells (117). TIGIT shows immunosuppressive functions by directly binding to tumor cells, which commonly express CD155, leading to T and NK cell inhibition (**Figure 1**). TIGIT acts also indirectly via stimulation of immunosuppressive DCs and Tregs after CD155/CD226 molecule recognition (128). Overexpression of TIGIT was associated with poor prognosis in many cancers (129). TIGIT expression is considered a marker of T cell exhaustion in liver cancer (130). Encouraging results presented in 2021 suggested that the combination of anti-TIGIT and anti-PD-L1 cancer immunotherapies could represent a novel approach in cancer (131). The recent failure of the tiragolumab trial was announced (132) in which tiragolumab was unable to demonstrate additional benefit in PFS over atezolizumab alone in a phase 3 trial in NSCLC patients. However, the data are still not mature and there are several other compounds in development across a range of indications, including EOS-448, vibostolimab, domvanalimab, ociperlimab, BMS-986207 or etigilimab, bringing the hope that a new class of checkpoint inhibitors would offer therapeutic benefits for cancer patients. TIGIT inhibition was assessed in 65 clinical studies at the beginning of 2023 (31).

###### 2.4.1.7.4 The role of CTLA-4 (CD152)

The anti-CTLA-4 antibody ipilimumab was the first immune checkpoint inhibitor approved in 2011 by the U.S. Food and Drug Administration for the treatment of late-stage melanoma (133), paving the way for the further research of immune checkpoint blockade. Recently, another CTLA-4 antagonist, tremelimumab, received priority review in the US FDA, supporting the combination of anti-CTLA4 antibody, tremelimumab, and the anti-PDL-1 antibody durvalumab for the treatment of patients with unresectable hepatocellular carcinoma. CTLA-4 and CD28 are co-receptors that bind to CD80 (B7-1) and CD86 (B7-2) to regulate T cell activation. CD28 co-stimulation is required for T cell activation, whereas CTLA-4 inhibits T cell responses by opposing the actions of CD28-mediated co-stimulation (**Figure 1**). CTLA-4 is highly expressed on activated and exhausted CD4 T cells, Tregs, activated and exhausted CD8 T cells, and in some tumor cells (134). A correlation has been observed between high levels of CTLA-4 expression and poor cancer prognosis (135). Blocking Treg function and the CTLA-4 pathway could constitute an effective synergistic mechanism to enhance antitumor activity by increasing the immune response. CTLA-4 blockade was assessed in 239 ongoing studies in different therapeutic combinations as of early 2023 (31).

###### 2.4.1.7.5 The role of PD-1 (CD279)

PD-1 is another membrane-bound co-inhibitory receptor expressed across hematopoietic and non-hematopoietic cells. The PD-1 receptor was described in the 1990s (136). PD-1 binds its ligands, PD-L1 and PD-L2 which are found on APCs, endothelial cells, cancer cells, mast cells and lymphocytes (137). PD-1 negatively regulates T cell-mediated responses via PD-L1 (138) (**Figures 1**, **2**). Moreover, PD-1 signaling can reduce secretion of IL-2, IFN-gamma, and TNF-alpha cytokines as well as reduce cell proliferation (139). PD-1-expressed on tumor-infiltrating T cells can bind to PD-L1 on the surface of cancer cells or other cells; blockade of PD-1 signaling is considered to be an effective way to restore T cell cytotoxic activity (140). Several IgG1 anti-PD-L1 antibodies, including atezolizumab (141) and avelumab (142) are able to induce cytotoxic or phagocytic effects, including antibody-dependent cellular cytotoxicity (ADCC), in addition to their PD-L1 blockade action. Initial studies targeting PD-1 and PD-L1 in advanced solid tumors allowed for the development of the first PD-1 inhibitors, nivolumab and pembrolizumab (143). Since the approval of pembrolizumab in 2014, the clinical development of PD-1 and PD-L1 inhibitors has been significantly widened. So far three PD-1 (pembrolizumab, nivolumab, and cemiplimab) and three PD-L1 (atezolizumab, avelumab, and durvalumab) inhibitors have been approved for cancer therapy, with numerous molecules in development (144). Lack of sustained response and development of resistance mechanisms remains a clinical issue during anti-CTL-A4 and anti-PD-1/anti-PD-L1 therapy. Key mechanisms underlying resistance to PD-L1 therapies include: loss of PD-L1 expression, the expression of soluble forms of the receptor, non-canonical WNT ligand-activation inhibiting T cell function, loss-of-function mutations in *JAK1/2* leading to the decreased expression of PDL-1 or activation of alternative immune checkpoints, e.g., TIM-3 and LAG-3 (145) (**Figure 1**). Anti-PD-1/anti PD-L1 blockade was assessed across different indications and combinations in 1665 studies at the beginning of 2023 (31).

#### 2.4.2 Humoral mechanisms

The immunosuppressive TME is influenced by several metabolic, humoral and regulatory pathways. A deeper understanding of these mechanisms enables the development of novel possibilities for therapeutic intervention. Some of these mechanisms and their importance are discussed below and shown on **Figures 1**, **2**.

##### 2.4.2.1 Cytokine shift into Th2 profile

Immunosuppression within the TME is characterized by a shift from a Th1 anti-inflammatory to a Th2 immunosuppressive cytokine profile (**Figures 1**, **2**). Cytokine-based therapies are being widely investigated in clinical trials – there were 805 registered studies assessing cytokines in different clinical settings at the beginning of 2023 (31).

##### 2.4.2.2 The role of chemokines in the TME

CTLs do not express chemokine receptors and therefore have difficulty infiltrating the TME. A recent proof of concept report showed promising results for a tumor re-programming therapy, which selectively enhanced local CTLs infiltration in patients with metastatic triple negative breast cancer. Patients received a chemokine-modulating regimen consisting of rintatolimod, selective TLR3 ligand, IFN-alpha, and concomitant therapy with cyclooxygenase-2 (COX-2) inhibitor celecoxib during their follow up pembrolizumab therapy. Significant increases of intratumoral type 1 immune antitumor markers upon treatment were observed, including granzyme B, ratios of CD8alpha/FOXP3 and granzyme/FOXP3, as well as CXCL10 and CCL5. In contrast, neither the Tregs marker Foxp3, nor Tregs attractants CCL22 or CXCL12 were enhanced. Three out of six patients had stable disease and an additional patient had a partial response (146). Chemokine based therapies are broadly assessed in clinical trials with 101 trials assessing chemokines in combination with other therapies (31)

##### 2.4.2.3 Inhibition of type 1 IFNs function

Type 1 IFNs are indispensable to the development of antitumor immunity by enhancing intratumoral CTL-DC crosstalk (147), and increasing of NK and M1 macrophages activity in the TME (148). Moreover, the efficacy of radiotherapy, chemotherapy and immunotherapy rely to a great extent on type 1 IFN signaling within tumors (149). Drugs inducing type 1 IFN responses are used widely as adjuvants for existing therapies (150). There is some evidence that type 1 IFN signaling also exerts a negative effect on antitumor immunity. Namely, chronic type 1 IFN signaling stimulates the immune response leading to an exhaustion state (151). These events lead to a defective pro-inflammatory cytokine production, adaptive resistance to therapy, and decreased activity of antigen-specific cells. In early 2023, there were 18 studies assessing type 1 IFN in cancer patients (31).

Moreover, in cancer, the cGAS-STING path appears to be a major innate immune pathway that can stimulate DC activation and T cell priming against tumor antigens due to stimulation of IFN genes. It has been shown, that radiotherapy-induced DNA damage leads to the formation of double-stranded DNA fragments recognized by cGAS in the cytosol. Indeed, irradiated tumor cells can activate this pathway stimulating the immune response and enhancing radiation efficacy (152, 153). Defects in cGAG/STING signaling induced by mutations, epigenetic control, or silencing, affect this mechanism and diminish the antitumor immune response in several malignancies (154). Therapeutic strategies engaging this pathway include the use of STING agonists (155). These modalities could be an attractive clinical approach to initiate *de novo* inflammation, DC activation, and T cell priming, especially in non-T cell-inflamed tumors. At the beginning of 2023, there were 13 trials assessing different combinations of immunotherapy with novel STING agonists in various indications, including TAK-500, or GSK3745417 (31).

#### 2.4.3 TME-dependent metabolic mechanisms

##### 2.4.3.1 The role of the NO/iNOS pathway

Along with arginase, iNOS is considered to be a regulator of immune suppression in the TME. However, the activation of these two regulators is competitive and depends on the polarization of the macrophages. M1 macrophages express iNOS, which metabolizes arginine to NO (nitric oxide) and citrulline, whereas M2 macrophages express arginase 1 and arginase 2 enzymes, which hydrolyze arginine to ornithine and urea. The M2 arginase pathway limits arginine availability for NO synthesis. The suppressive effects of NO on T cell function are mediated by the inhibition of the JAK3/STAT5 pathway, the reduction of MHC class II expression, and the induction of T cell apoptosis (156). Furthermore, NO can induce T cell anergy (157)and recruit MDSCs, Tregs, M2 macrophages and Th2 cells to the TME, to develop “cold” tumors (97). Additionally, NO inhibits the production of IL-12 by DCs and M1 macrophages (158). At the beginning of 2023 there were 13 studies targeting the NO/iNOS pathway in cancer patients (31).

##### 2.4.3.2 Impact of arginase-1 and arginase-2 pathway

This pathway promotes the catabolism of arginine into urea and ornithine in tumors, which further utilize these metabolites for collagen biosynthesis (159). M2 macrophages and MDSCs are considered the regulators of arginine metabolism in the TME via ARG1 expression (160). The expression of this enzyme is increased in response to Th2 and immunosuppressive cytokines (e.g., IL-4, IL-13, IL-10, TGF-beta) contributing to the resolution of inflammation. Deprivation of arginine has a negative effect on tumor growth via autophagy, apoptosis, and cell cycle arrest (161). In addition, it decreases T cell signaling, proliferation, and IFN-gamma production (162). ARG1 expression by MDSCs favors the generation of IDO1-expressing, immunosuppressive DCs (163). The inhibition of ARG1 and ARG2 activity has shown positive results across numerous cancer models by reducing myeloid-driven immune suppression (164), however, there were only 2 studies assessing ARG1 and ARG2 in cancer patients at the beginning of 2023 (31).

##### 2.4.3.3 The role of the adenosine pathway

The cell surface ectonucleotidases, CD39 and CD73 regulate the conversion of extracellular adenosine triphosphate (eATP) to adenosine. Elevated levels of hypoxia-inducible factor -1 alpha (HIF-1 alpha), IL-1 beta, IL-6, TNF-alpha, TGF-beta were shown to raise CD39 and CD73 levels (165) (**Figure 1**). Adenosine supports immunosuppression via the adenosine A1 receptor (A1R), adenosine A2A (A2AR), adenosine A2B (A2BR) and adenosine A3 (A3R) receptors (166) expressed on immune cellss (167), and some tumor cells. The A2A receptor promotes the proliferation and immunosuppressive function of Tregs (168) while inhibiting CTLs proliferation, cytotoxicity and ant-tumor cytokine production (169). High adenosine level may stimulate macrophage differentiation into M2 phenotype and enhance their VEGF, IL-6 and IL-10 synthesis (170). Another population of highly immunosuppressive cells, MDSCs, produces extracellular adenosine in the TME after TGF-beta-induced expression of CD39 and CD73 (171). Additionally, adenosine can stimulate the accumulation of MDSCs within TME and promote MDSCs expansion (172). Moreover, activation of A2AR on neutrophils, M1 macrophages, NK cells, Th1 cells and DCs inhibited antitumor cytokines and chemokines production (173). Finally, the adenosine stimulated cancer cells result in enhanced proliferation, migration and metastasis in enhanced proliferation, migration and metastasis (174). With all this data, blocking adenosine signaling is considered to be a feasible approach to change the immunosuppressive TME. Clinical trials targeting the A2A receptor in patients with refractory renal cell cancer and other indications are in progress (175). A completed phase 1 study in metastatic castration resistant prostate cancer (mCRPC) showed that mCRPC can be sensitive to A2AR blockade with ciforadenant. Furthermore, the cytokine changes observed provided evidence of treatment-induced inflammatory response (176). The potential advantage of this therapy is, that it is suitable for combination with other anti-adenosine agents targeting the pathway at a different level (e.g., A2AR with anti-CD73), and with other types of immunotherapies. The main limitations of these agents are the short half-lives, limited efficacy in monotherapy, and uncertainty regarding the best combination approaches. At the beginning of 2023, the blocking of the adenosine pathway was assessed in 85 clinical trials (31).

##### 2.4.3.4 The role of IDO1/tryptophan and the kynurenine pathway

IDO1 is an enzyme catalyzing tryptophan, in the initial step of the kynurenine pathway. IDO1 is expressed by tumor cells, tolerogenic DC cells, MDSCs cells and fibroblasts (177). Tryptophan deprivation leads to T cell cycle arrest and induces T cell anergy (178) (**Figure 1**). Its immunosuppressive catabolite, kynurenine, mediates the differentiation of CD4+ T cells into Tregs (179), and inhibits CTLs survival and proliferation (180). Kynurenine was also reported to dampen NK cell function and proliferation (181). Drugs targeting the IDO1 pathway are currently in early-phase clinical trials or in preclinical development. IDO1 pathway-inhibiting drugs in trials include indoximod, NLG919 and INCB024360. Ongoing trials combine indoximod with conventional chemotherapy. Other trials assess the combination of INCB024360 or indoximod with checkpoint inhibitor therapies. There were 23 studies investigating agents blocking the IDO1 pathway at the beginning of 2023 (34).

##### 2.4.3.5 Hypoxia-associated mechanisms

The presence of hypoxic conditions in the TME is associated with rapid tumor growth and influences significantly the immune status within the tumor (**Figure 1**). The relationship between hypoxia and immune suppression in the TME is linked to an impairment of type 1 IFN signaling, upregulation of immune checkpoint molecules, and the extracellular TGF-beta and adenosine levels (182). The response to hypoxia is driven by HIF-1 alpha, HIF-2 alpha, HIF-3 alpha, which are oxygen-sensitive transcription factors. One of the most important immunosuppressive mechanisms promoted by hypoxic conditions is the effect on TGF-beta levels. It has been shown, that under hypoxic conditions, the HIF-1 alpha level correlated with the activated TGF-beta signaling pathway (183). Moreover, HIF-1 alpha-mediated the switch in TGF-beta function from inhibiting to promoting glycolysis (184). Additionally, HIF-1 alpha-dependent induction of FoxP3, a key transcriptional regulator for Tregs was sufficient to drive Tregs abundance and activity (185). This implies, that increased levels of TGF-beta could accentuate the immunosuppressive impact of Tregs in the TME. An additional link between the PI3K/AKT/mTOR pathway and hypoxia can promote metabolic reprogramming of tumor cells. This process of aerobic glycolysis called the Warburg effect (186) relies on the predominant diversion of pyruvate to lactate. Although the genetic events leading to the Warburg effect are not fully known, the PI3K/AKT/mTOR pathway plays an important role in this process. Activation of AKT promotes aerobic glycolysis (187) and expression of constitutively activated AKT results in a growth factor-independent increase in glucose uptake and glycolytic rate (188). The release of lactic acid contributes to acidity, which further promotes the recruitment of Tregs into the TME. This effect further suppresses anticancer immunity and represents one of the main causes of anticancer immunotherapy failure (189). Hypoxia-activated prodrugs are designed to target tumor cells resistant to conventional therapies. Evofosfamide and tarloxotinib are currently in active clinical development. A different approach targeting the HIF pathway include the HIF-2 allosteric inhibitor belzutifan (190). At the beginning of 2023, there were 102 trials registered targeting hypoxia in cancer patients (31).

#### 2.4.4 Other mechanisms

##### 2.4.4.1 The role of PGE2

PGE2 is a lipid derivative generated by the effects of the enzyme COX-2 following the enzymatic conversion from arachidonic acid. In the TME, PGE2 is synthesized by myeloid, stromal and cancer cells (191). In cancer, PGE2 is considered as a key immunosuppressive mediator inhibiting CTLs, NK cells and the type 1 inflammation response, while promoting Tregs, MDSCs expansion and type 2 inflammation (**Figure 1**) (192). Targeting the production, degradation and responsiveness to PGE2, provides tools to modulate the patterns of immunity in a wide range of malignancies. There were 11 ongoing studies investigating the inhibition of this pathway in cancer patients reported at the beginning of 2023 (31).

##### 2.4.4.2 The emerging role of extracellular vesicles

Extracellular vesicles (EVs) consist of variety of subtypes, including: exosomes, microvesicles, ectosomes, oncosomes, and apoptotic bodies (193). Exosomes are nanosized vesicles actively secreted by fibroblasts as well as endothelial, epithelial, neuronal, immune and cancer cells (194). Exosomes secreted by tumor cells can play important roles in cancer progression and invasion, including TME remodeling, tumor metastasis and tumor-associated immunosuppression (195). Tumor cells can release growth factors, glycans, lipids, metabolites, microRNAs (miRNA) (196) and DNA (197) as soluble molecules but also encapsulated or bound to extracellular vesicles (198). Tumor-derived EVs can contain immunosuppressive molecules such as PD-L1, TGF-beta 1, FasL, TRAIL, and NKG2D ligands, which make them important mediators of tumor immune evasion (199). Among the different types of EVs, one group is classified as small extracellular vesicles (sEVs). These are small membrane vesicles of a diameter approximately 100 nm (200). sEV might be involved in genetic exchange between cells by transfer of mRNAs and miRNAs (201). They may be also engaged in remodeling the ECM of pre-metastatic niches and facilitate the formation of immunosuppressive environments in distant organs. Exosomal PD-L1 may become targets for anti-PD-1/PD-L1 antibody therapy and chemotherapeutic drug carriers (202) helping to reprogram the immunosuppressive TME. Recently, it was shown that PD-L1 levels from EVs predict a durable response to immune checkpoint inhibitors and survival in patients with NSCLC (203). Several production and pharmacokinetic challenges have to be overcome to enable wide therapeutic usage of sEVs (204). The FDA has not approved to date any exosome products, but exosome based therapies were investigated in 50 clinical trials in cancer patients in the beginning of 2023 (31).

#### 2.4.5 Tumor intrinsic immune escape mechanisms

Cancer cells express various cytokines, chemokines, and growth factors. These include, but are not limited to IL-6, IL-8, CCL2, M-CSF, granulocyte-macrophage colony-stimulating factor (GM-CSF), CXCL10, CXCL12, VEGF-A, TGF-beta and G-CSF (205) among others. These molecules contribute to a variety of functions related to systemic inflammation and cancer progression (**Figure 1**). Another way of influencing tumor cell-dependent immune escape mechanisms is offered via epigenetics. Several studies revealed a pivotal role of epigenetics in tumor cell regulation. Epigenetic mechanisms in the TME are involved in the upregulation of IL-6 and G-CSF and the downregulation of CXCL9 and CXCL10 via EZH2. These changes can increase MDSCs recruitment into the TME and decrease T cells and DC infiltration (206). It has been shown, that the expression of CCL2 and CCL20 could be increased by miRNA molecules (207). This promotes immune escape, as CCL2 enhances the recruitment of TAMs and Tregs into the TME. Increased expression of CCL20 plays a role in the recruitment of Th17 cells. Moreover, tumor cells can secrete TGF-beta (**Figures 1**, **2**), which suppresses M1 macrophages, NK cells, DCs and T cells immunity by regulating the expression of miRNAs in tumor and NK cells (208). Another way of immune escape is the expression of immune checkpoint molecules by cancer cells such as PD-1, PD-L1, lymphocyte-activation gene 3 (LAG3), TIM3, T cell immunoreceptor with Ig and ITIM domains (TIGIT), V-domain Ig suppressor of T cell activation (VISTA) and human leukocyte antigen G (HLA-G) (**Figure 1**). Immune checkpoint mechanisms help to maintain self-tolerance and protect against auto-immunity in physiological conditions. However, in tumorigenesis, these mechanisms are adopted by tumors to achieve immune escape (209), as discussed below and as presented on **Figures 1** and **2**.

##### 2.4.5.1 The emerging role of the glycocode

Many authors indicate that cancer-induced glycan signatures called the “glycocode” could be considered a novel type of immune checkpoint (210) (**Figure 1**). Cancer transformation causes altered glycosylation processes within tumor cells and within the TME. One of such modifications is the expression of altered glycan structures or lectin receptors on the cancer cell surface. Modified glycan structures can bind the lectin receptors expressed on monocytes, macrophages, DCs, TAMs and NK cells. Examples of such modified glycan structures include sialic acid end-standing glycans, N-acetylgalactosamine glycans (GalNAc) or Lewis X glycan (211). Altered glycan responses can promote immune suppression by modification of antigen-presenting cell functions, increasing differentiation of M2 TAMs, diminishing CTLs differentiation and decreasing NK cells activity (212). This results in enhanced immune evasion within the TME, including stimulation of immunosuppressive cytokines, decreased secretion of inflammatory cytokines (213), and the induction of Th2 cells (214) and Tregs (215). Changes in the metabolism of glycans can be regulated by transcription factors, genetic and epigenetic changes or an altered metabolism contributing to tumor cell proliferation and invasiveness (216). Moreover, altered glycosylation of tumor proteins can create cancer neo-antigens, which can be recognized by tumor-specific cytotoxic T cells (217). The first clinical attempts at targeting the glycocode in the TME showed encouraging results. Improved analytical methods and the development of novel strategies for targeting the tumor glycocode antigens could present a promising therapeutic strategy in the future.

##### 2.4.5.2 Metabolic reprogramming – a hallmark of cancer

Metabolic reprogramming appears to be a key immunosuppressive mechanism within the TME ^293^ (**Figures 1**, **2**). The most characteristic feature of metabolic reprogramming in cancer cells is the induction of hypoxia, in addition to the existing hypoxia present in TME, already discussed in one of the previous sections. In summary, in cancer cells hypoxia regulates the expression of multiple key genes involved in immunosuppression via HIF-1 alpha, TGF-beta secretion, increase of EMT, and the activation of signaling pathways enhancing the recruitment of MDSCs and Tregs into TME (218). Moreover, hypoxia impacts metabolic reprogramming via the PI3K/AKT/mTOR pathway. It has been shown that mTOR (mTORC1) regulates the expression of HIF-1alpha (219), which can stimulate glucose uptake via enhancing the expression of glucose transporters and glycolytic enzymes (220). As we already mentioned above, oxidative glycolysis, known as the Warburg effect, provides substrates for metabolic pathways to produce protein, lipids, and nucleotides required for cell growth and proliferation (221). The activity of the CDK8 kinase is also considered a significant factor for metabolic reprograming as it regulates the glucose transporter expression, glucose uptake, glycolytic processes, as well as cell cancer proliferation and growth, both in normoxia and hypoxia (222). Reprogrammed pathways aid supporting the needs of rapid cell proliferation, survival, migration, metastasis and resistance to cancer treatments (223). In addition to the metabolic reprogramming of tumor cells and immune cells in TME, the metabolism of the gut microbiome has recently gained increasing attention on the anti-tumor immune regulation. Microbiota-derived short-chain fatty acids, such as sodium butyrate, promote the formation of memory T cells and modulate Tregs function (224). Moreover, sodium butyrate promotes the proliferation of normal colon cells and serving as a histone deacetylase inhibitor, epigenetically suppresses the proliferation of cancerous colon cells undergoing the Warburg effect (225).

##### 2.4.5.3 The role of epigenetic mechanisms

Epigenetics examines mechanisms modifying the expression of genes, without changing the DNA nucleotide sequence, reversibly, heritably and adaptively (226). Epigenetic changes in genes encoding tumor suppressors or antitumor cytokines, could lead to an impaired anti-cancer immunity, immune escape, and drug resistance, which results in tumor growth, progression and metastasis (227). The best known epigenetic mechanisms responsible for these processes are modifications of histone marks and chromatin structures, alteration of DNA methylation and changes in miRNA expression levels (228). The importance of epigenetic mechanisms in cancer led to the development of new molecules used as anticancer therapies (229).

###### 2.4.5.3.1 Epigenetic modifications of histones and modifications of chromatin structures

These include fixation to DNA of methyl groups and chemical histone-post translational modifications. Histone-post translational modifications can influence the chromatin structure via the recruitment of regulatory proteins, and/or altering the charge of histones (through acetylation). Histone deacetylases (HDACs), histone methyltransferases (HMTs) and the family of bromodomain and extra-terminal domain (BET) proteins, all three seem to be the most involved factors in the cancerogenesis process (230) involving epigenetic modifications, and as such, they are the target of cancer therapies: inhibitors of histone deacetylases (HDACi), histone methyltransferase inhibitors (HMTi), and histone reader protein inhibitors (bromodomain and extra-terminal domain proteins – BETi).

###### 2.4.5.3.2 The role of HDACi

HDAC inhibitors can reduce tumor growth and promote apoptosis (231). Treatment with HDACi was shown to increase the expression of MHC-I molecules on tumor cells and the expression of tumor antigens, enhancing the effects of cytotoxic lymphocytes (232). HDACi can also increase the NK cell activity by increasing the upregulation of NKG2D ^397^. It has been shown that HDAC inhibitors can restore TP53 protein transcription and allow resistant cancer cells to undergo apoptosis (233). There were 68 trials reported assessing HDAC inhibitors in different oncology indications early in 2023 (34).

###### 2.4.5.3.3 The role of HMTi/inhibitors of EZH2

EZH2 is a chromatin mark involved in gene silencing and developmental regulation (234). Overexpression of EZH2 has been observed in breast cancer, bladder cancer, prostate cancer and melanoma (235). EZH2 is also activated in lymphomas through several mutations (236). EZH2 inhibitors restore the secretion of Th1-type chemokines, increase CTLs-tumor infiltration, inhibit tumor progression, and they can improve the efficacy of anti-PD-L1 agents (237). EZH2 inactivation reversed the resistance to anti-CTLA-4 and IL-2 immunotherapy and suppressed melanoma growth in mice models (238). There were 22 trials assessing EZH2 inhibitors in different cancer indications early in 2023 (31).

###### 2.4.5.3.4 The role of BETi

Histone reader proteins bind to the histone structure and interpret the histone code into functional outcomes. The BET family of proteins are histone reader proteins binding acetylated histones and modulating immune-response gene transcription (239). In cancer cells, inhibition of the BET family reduces cytokine production, NFκB activity, PD-L1 expression, and increases natural killer cell-activating ligands (240). Furthermore, BET proteins regulate chromatin remodeling and promote tumor-associated inflammation. The inhibition of BET proteins stimulates an anti-inflammatory effect within the TME (241). Bromodomain proteins are considered an attractive target for anticancer treatments. At the beginning of 2023 Bromodomain proteins targeting drugs were assessed in 18 clinical trials (31).

###### 2.4.5.3.5 Impact of DNA methylation

This process relies on the addition of a methyl group to cytosine bases creating 5-methylcytosine at CpG sequences in gene-promotor regions (242). These DNA methylation marks block transcription, lead to long-term transcription repression and they are associated with gene silencing. DNA methylation is carried out by DNA methyltransferase enzymes. DNA methyltransferase inhibitors (DNMTi) increase expression of tumor antigens through the enhanced expression of MHC molecules and tumor antigens (243). In addition, DNMTi can reactivate retroviruses normally suppressed by DNA methylation in somatic cells (244). This increases the recruitment of CTLs in the TME, the stimulation of antitumor cytokine production, and it can also increase IFN signaling (245). Targeting DNA methylation and EZH2 activity can overcome melanoma resistance to immunotherapy via modulating PD-L1 expression and/or T cell infiltration (246). Azacitidine, decitabine or guadecitabine (247) with the newer molecule CC-486, are examples of DNMTi drugs which are used in combination with immunotherapy, and they could provide additional benefits to patients with low PDL-1 expression (248). At the beginning of 2023, there were 375 trials assessing these compounds in different cancer indications and combinations (31).

###### 2.4.5.3.6 Emerging role of miRNAs

miRNAs are single-stranded, noncoding small ribonucleic acid (RNA) fragments. They can negatively regulate gene expression at the posttranscriptional level (249). Pairing of miRNA with a target messenger RNA (mRNA) can lead to the inhibition of translation and to mRNA degradation (250). MiRNA-based drugs (miRNA mimics or miRNA antagonists) are considered to be a promising strategy for cancer therapy (251). There were 123 trials assessing miRNA in different cancers early in 2023 (31).

#### 2.4.6 Stroma dependent mechanisms

As described previously, stromal factors contribute to the immunosuppressive TME (20). The tumor stroma consist mainly of cancer-associated fibroblasts (CAFs), endothelial cells, cancer associated adipocytes (CAAs) and multipotent stem cells (MSCs); in addition, collagen bundles and dense ECM characterize this milieu with poor oxygen and nutrient availability (252).

CAFs contribute to tumor immune escape by promoting cancer cell proliferation via the secretion of growth factors, the induction of angiogenesis and through the remodeling of the ECM, which supports tumor cell invasion (253). CAFs mediate tumor-promoting inflammation via the secretion of cytokines and chemokines, which in turn enhance the recruitment of immune cells (254). Endothelial cells (ECs) constitute another subpopulation of stromal cells (255). Tumor-associated ECs (TECs) form the vascular inner layer of tumors (256). TECs are known to be particularly important for T cell recruitment and activation, tumor cell growth and invasion (257), as well as influencing antitumor cell immune responses.

CAAs play an important role in tumorigenesis, tumor growth, and metastasis (258). CAAs can support cancer cells by storing energy as triacylglycerol and act as a source of lipids. Another population of stromal cells in cancers are MSCs, which are found in most cancers playing a central role in tumor growth, invasion, and metastasis. These cells are able to interact directly with tumor and immune cells in the TME (259). A dense stroma inhibits the infiltration of immune cells into the TME (**Figure 1**). It was been shown that the immature myxoid stroma was associated with lower densities of tumor intraepithelial memory cytotoxic T cells and stromal M1-like macrophages (260). Collagen density is relatively large in the TME, which can affect the phagocytotic ability of macrophages (261). In addition, an increase in interstitial pressure in the stroma due to hyaluronan deposits contributes to the inhibition of immune cell penetration into the TME, posing a mechanism of resistance to immunotherapies and a sign of poor disease prognosis in some indications such as pancreatic cancer (**Figure 1**).

Targeting the pro-fibrotic function of CAFs in clinical settings was performed using pirfenidone, an antifibrotic agent and TGF-beta antagonist, as well as tranilast. It was noted that the antitumor effects were enhanced when targeting CAFs in combination with effector-stimulatory immunotherapy such as dendritic cell-based vaccines (262). CAFs targeted therapies are being assessed in 63 clinical trials (31).

## 3 Therapeutic possibilities targeting multiple immunosuppression mechanisms in the TME

### 3.1 The role of polyspecific antibody combinations

Polyspecific monoclonal antibodies (PsMabs) are genetically engineered proteins that can simultaneously engage two or more different types of epitopes (263). They show several advantages over monoclonal antibodies. They can redirect specific polyclonal immune cells such as T and NK cells to tumors and simultaneously block two different pathways with unique or overlapping functions in pathogenesis. This reduces the cost of development in comparison to multiple single epitope-based antibodies in combination therapy, or compared to the production of CAR-Ts (264). PsMabs antibodies may also be conjugated to biomaterials or nanoparticles, to achieve prolonged, local release (265). Improving antibodies by pH activation and glycosylation (266) could enhance the specificity and potency of immunotherapies and limit unwanted toxicity.

### 3.2 Vaccines as a promising tool to overcome immunosuppression

Anticancer vaccines can be divided into four categories: cell-based vaccines, peptide-based vaccines, viral-based vaccines, and nucleic acid-based vaccines (267). Cell-based cancer vaccines are prepared from whole cells or cell fragments, containing tumor antigens and inducing a broad antigen immune response. DCs vaccines is an example of a cell-based vaccine category (268). Personalized neoantigen cancer vaccines based on DCs showed a promising antitumor effect. However, long preparation process and the high cost are factors limiting development of these type of vaccines. Engineered virus vaccines can carry tumor antigens to activate the immune response. In the case of oncolytic viruses, they can lyse the tumor cells, releasing additional tumor antigens, further increasing the vaccine’s effectiveness, and contributing to long-term immune memory. Again, the clinical application of these vaccines is limited by the complex preparation process. Peptide-based vaccines induce a robust immune response against tumor antigens. For example, virus-like particles (VLP) vaccines, containing viral protein complexes that mimic the native virus structure without being infectious, showed promising antitumor activity activating cellular immune responses (269). Nucleic acid vaccines induce strong MHC-I mediated CTLs responses (270). Nucleic acid vaccines can simultaneously deliver multiple antigens to trigger humoral and cellular immunity. Additionally, they can encode full-length tumor antigens, allowing antigen presenting cells (APCs) to present various epitopes and antigens simultaneously. The nucleic acid vaccine preparation is simple and fast, which facilitate development of personalized anticancer vaccines. Nanoparticle systems have shown promising results as delivery vectors for anticancer vaccines in preclinical research. In addition, neo-antigen vaccines in combination with checkpoint blockade therapies using anti-PD-1 and anti-PD-L1 antibodies showed potent therapeutic effects in patients with advanced cancer, however this therapy is in the early stages of development (271). Early in 2023, there were 495 clinical trials assessing anticancer vaccines (31).

### 3.3 Radiation as an additional tool to stimulate anti-tumor responses

The immunosuppressive TME contributes to poor antigen presentation and protects the tumor from immune defense. Radiation may reveal tumor antigens, thus modifying the TME and improving innate and adaptive immune responses (272). Radiation therapy can promote an immunogenic form of cell death stimulating the activation of a tumor-targeting immune response (273) and it is frequently used in combination with other forms of targeted therapy or immunotherapy. There were more than 4000 trials assessing usage of radiotherapy and radiation in cancer patients in different regimens and settings at the beginning of 2023 (31).

### 3.4 The emerging role of nanomedicine and nanoparticle applications

Nanomedicine can be defined as a use of nanotechnology materials of a size between 1 and 200 nm for medical purposes (274). Nanomedicines, due to their small size, plasticity, and heterogeneous properties, can selectively reach the tumor tissue, they can be used as carriers of drugs, to improve bioavailability and to extend the half-life of molecules, or they can be used to release therapeutic agents in answer to stimuli. Examples of nanomedicines undergoing research are: lipid-based (liposome, solid lipids, stealth liposomes), polymer-based, inorganic (metal, silica, hafnic oxide nanoparticles), viral, and drug-conjugated nanoparticles (antibody drug conjugates, polymer drug conjugates, polymer protein conjugate). Nanocarrierrs can change the properties of compounds, for example coating the nanoparticles with polyethylene glycol (PEG), a hydrophilic and non-ionic polymer, increases their solubility and stability (275). Several formulations are approved for clinical usage, including liposomal danorubicin and doxorubicin, nanoparticle albumin bound paclitaxel, liposomal PEGylated irinotecan, polyethylene glycol–polylactide (PEG–PLA), polymeric micelle (276). Nanoparticles could be delivered to the tumor tissue in several ways. Passive targeting relies on the leaky vasculature within tumors, allowing nanoparticles to reach cancer cells via the fenestrations. Active targeting uses ligands on nanoparticles’ surface which recognize and bind receptors overexpressed on tumor cells. Triggered release allows nanoparticles to act if exposed to an external stimulus such as a magnetic field or light. Changes in pH, redox, ionic strength, and stress in target tissues are examples of internal stimuli ^306^. An emerging method is the use of theranostics, which combines both, the ability to diagnose and treat cancers. In theranostics, not only can the release of the drug be monitored, but the effects of the drugs in the tumor tissue can also be visualized (277). Nanoparticles could improve the administration and efficacy of immunotherapies and contribute to the further progress of cancer treatment. Nanoparticles are able to overcome physical and biological obstacles in the delivery of immunomodulating therapies to the TME, and they are able to modify the TME to increase tumor immune infiltration (278). The exceptional heterogeneity of nanoparticles places this field at the top of the research interest, with 54 trials assessing nanoparticles in clinical settings (31).

## 4 Conclusions

The immune host response can effectively eliminate cancer cells, or on the contrary, support cancer growth. The final outcome of the immune response depends on mutual interactions between immune, stromal, and tumor cells involved in the TME. Numerous immune escape mechanisms have been identified within tumors. These mechanisms can be associated with tumor, immune, and stromal cells, and they can present a variety of components including humoral, metabolic, epigenetic, and genetic factors among others. The knowledge of tumor-escape mechanisms enables targeted interventions, as well as the implementation of combination therapies to overcome them. However, the limitations in the use of combination therapies depend on the onset of adverse effects and toxicities in patients, limiting the therapeutic efficacy of these combinations. These limitations pose a clear barrier to the use of newly-discovered drugs, able to counteract pro-tumorigenic pathways. Therefore, additional efforts in oncology research and clinical development strategies should be implemented in the future to mitigate drug toxicities, and to enable more complex combinations of therapeutic agents.

As shown in this article, the application of modern technologies, including nanomedicines, ICIs, adoptive cell, and epigenetic therapies can in some situations reprogram the TME and shift the host response into an antitumor response. These events translate into series of breakthroughs in cancer therapies currently observed in clinical practice. As we mentioned above, joined efforts between scientists and clinicians offer the potential to create even greater hopes for the identification and clinical application of new TME-targeting drugs in the near future while maintaining low toxicities.

## Author contributions

PC-F, MPr and KS conceptualized the manuscript, PC-F wrote the first draft of the manuscript. All authors contributed to the article and approved the submitted version.

## References

[1] Elomaa H Ahtiainen M Väyrynen SA Ogino S Nowak JA Friman M. Prognostic significance of spatial and density analysis of T lymphocytes in colorectal cancer. Br J Cancer (2022) 127:514–23. doi: 10.1038/s41416-022-01822-6 PMC9345858 35449453

[2] Väyrynen JP Haruki K Lau MC Väyrynen SA Ugai T Akimoto N. Spatial organization and prognostic significance of NK and NKT-like cells via multimarker analysis of the colorectal cancer microenvironment. Cancer Immunol Res (2022) 10(2):215–27. doi: 10.1158/2326-6066.CIR-21-0772 PMC8816895 34937729

[3] Yost KE Chang HY Satpathy AT. Recruiting T cells in cancer immunotherapy. Science (2021) 372(6538):130–1. doi: 10.1126/science.abd1329 33833111

[4] Hegde PS Karanikas V Evers S. The where, the when, and the how of immune monitoring for cancer immunotherapies in the era of checkpoint inhibition. Clin Cancer Res (2016) 22(8):1865–74. doi: 10.1158/1078-0432.CCR-15-1507 27084740

[5] Saini KS Punie K Twelves C Bortini S de Azambuja E Anderson S. Antibody-drug conjugates, immune-checkpoint inhibitors, and their combination in breast cancer therapeutics. Expert Opin Biol Ther (2021) 21(7):945–62. doi: 10.1080/14712598.2021.1936494 34043927

[6] Mariathasan S Turley SJ Nickles D Castiglioni A Yuen K Wang Y. TGF-beta attenuates tumour response to PD-L1 blockade by contributing to exclusion of T cells. Nature (2018) 554(7693):544–8. doi: 10.1038/nature25501 PMC6028240 29443960

[7] Tahkola K Ahtiainen M Mecklin JP Kellokumpu I Laukkarinen J Tammi M. Stromal hyaluronan accumulation is associated with low immune response and poor prognosis in pancreatic cancer. Sci Rep (2021) 11:12216. doi: 10.1038/s41598-021-91796-x 34108626 PMC8190291

[8] Liu Y-Q Zou H-Y Xie J-J Fang W-K. Paradoxical roles of desmosomal components in head and neck cancer. Biomolecules (2021) 11:914. doi: 10.3390/biom11060914 34203070 PMC8234459

[9] Pai SI Cesano A Marincola FM. The paradox of cancer immune exclusion: immune oncology next frontier. Cancer Treat Res (2020) 180:173–95. doi: 10.1007/978-3-030-38862-1_6 PMC7423459 32215870

[10] Shi X Young CD Zhou H Wang X. Transforming growth factor-β signaling in fibrotic diseases and cancer-associated fibroblasts. Biomolecules (2020) 10(12):1666. doi: 10.3390/biom10121666 33322749 PMC7763058

[11] Armitage JD Newnes HV McDonnell A Bosco A Waithman J. Fine-tuning the tumour microenvironment: current perspectives on the mechanisms of tumour immunosuppression. Cells (2021) 10(1):56. doi: 10.3390/cells10010056 33401460 PMC7823446

[12] Dzobo K Senthebane DA Thomford NE Rowe A Dandara C Parker MI. Not everyone fits the mold: intratumor and intertumor heterogeneity and innovative cancer drug design and development. Omics J Integr Biol (2018) 22:17–34. doi: 10.1089/omi.2017.0174 29356626

[13] Dzobo K Senthebane DA Rowe A Thomford NE Mwapagha LM Al-Awwad N. Cancer stem cell hypothesis for therapeutic innovation in clinical oncology? taking the root out, not chopping the leaf. Omics J Integr Biol (2016) 20:681–91. doi: 10.1089/omi.2016.0152 27930094

[14] Thankamony AP Saxena K Murali R Jolly MK Nair R. Cancer stem cell plasticity–a deadly deal. Front Mol Biosci (2020) 7:79. doi: 10.3389/fmolb.2020.00079 32426371 PMC7203492

[15] Prasetyanti PR Medema JP. Intra-tumor heterogeneity from a cancer stem cell perspective. Mol Cancer (2017) 16:41. doi: 10.1186/s12943-017-0600-4 28209166 PMC5314464

[16] van Niekerk G Davids LM Hattingh SM Engelbrecht A-M. Cancer stem cells: a product of clonal evolution? Int J Cancer (2017) 140:993–9. doi: 10.1002/ijc.30448 27676693

[17] Davis A Gao R Navin N. Tumor evolution: linear, branching, neutral or punctuated? biochim. Biophys Acta (2017) 1867:151–61. doi: 10.1016/j.bbcan.2017.01.003 PMC5558210 28110020

[18] Gilson P Merlin J-L Harlé A. Deciphering tumour heterogeneity: from tissue to liquid biopsy. Cancers (2022) 14:1384. doi: 10.3390/cancers14061384 35326534 PMC8946040

[19] Teng MW Galon J Fridman WH Smyth MJ. From mice to humans: developments in cancer immunoediting. J Clin Invest (2015) 125(9):3338–46. doi: 10.1172/JCI80004 PMC4588291 26241053

[20] Maleno I López-Nevot MA Cabrera T Salinero J Garrido F. Multiple mechanisms generate HLA class I altered phenotypes in laryngeal carcinomas: high frequency of HLA haplotype loss associated with loss of heterozygosity in chromosome region 6p21. Cancer Immunol Immunother (2002) 51:389–96. doi: 10.1007/s00262-002-0296-0 PMC11032975 12192539

[21] McEvoy CR Seshadri R Morley AA Firgaira FA. Frequency and genetic basis of MHC, beta-2-microglobulin and MEMO-1 loss of heterozygosity in sporadic breast cancer. Tissue Antigens (2002) 60(3):235–43. doi: 10.1034/j.1399-0039.2002.600305.x 12445306

[22] Kincaid EZ Che JW York I Escobar H Reyes-Vargas E Delgado JC. Mice completely lacking immunoproteasomes show major changes in antigen presentation. Nat Immunol (2012) 13:129–35. doi: 10.1038/ni.2203 PMC3262888 22197977

[23] Sade-Feldman M Jiao YJ Chen JH Rooney MS Barzily-Rokni M Eliane JP. Resistance to checkpoint blockade therapy through inactivation of antigen presentation. Nat Commun (2017) 8:1136. doi: 10.1038/s41467-017-01062-w 29070816 PMC5656607

[24] Ito S Okano S Morita M Saeki H Tsutsumi S Tsukihara H. Expression of PD-L1 and HLA class I in esophageal squamous cell carcinoma: prognostic factors for patient outcome. Ann Surg Oncol (2016) 23:508–15. doi: 10.1245/s10434-016-5376-z 27380638

[25] Dhatchinamoorthy K Colbert JD Rock KL. Cancer immune evasion through loss of MHC class I antigen presentation. Front Immunol (2021) 12:636568. doi: 10.3389/fimmu.2021.636568 33767702 PMC7986854

[26] Anfossi N André P Guia S Falk CS Roetynck S Stewart CA. Human NK cell education by inhibitory receptors for MHC class, I. Immunity (2006) 25:331–42. doi: 10.1016/j.immuni.2006.06.013 16901727

[27] Lee J-C Lee K-M Kim D-W Heo DS. Elevated TGF-β1 secretion and down-modulation of NKG2D underlies impaired NK cytotoxicity in cancer patients. J Immunol (2004) 172:7335–40. doi: 10.4049/jimmunol.172.12.7335 15187109

[28] Spel L Boelens JJ van der Steen DM Blokland NJG van Noesel MM Molenaar JJ. Natural killer cells facilitate PRAME-specific T-cell reactivity against neuroblastoma. Oncotarget (2015) 6:35770–81. doi: 10.18632/oncotarget.5657 PMC4742140 26452036

[29] Lee JH Shklovskaya E Lim SY Carlino MS Menzies AM Stewart A. Transcriptional downregulation of MHC class I and melanoma de- differentiation in resistance to PD-1 inhibition. Nat Commun (2020) 11:1–12. doi: 10.1038/s41467-020-15726-7 32312968 PMC7171183

[30] Lauss M Doniam M Harbst K Andersen R Mitra S Rosengren F. Mutational and putative neoantigen load predict clinical benefit of adoptive T cell therapy in melanoma. Nat Commun (2017) 8:1–11. doi: 10.1038/s41467-017-01460-0 29170503 PMC5701046

[31] Available at: www.clinicaltrials.gov (Accessed 7th February 2023).

[32] Tang M Diao J Cattral MS. Molecular mechanisms involved in dendritic cell dysfunction in cancer. Cell Mol Life Sci (2017) 74(5):761–76. doi: 10.1007/s00018-016-2317-8 PMC11107728 27491428

[33] Hargadon KM. Tumor-altered dendritic cell function: implications for anti-tumor immunity. Front Immunol (2013) 11:192. doi: 10.3389/fimmu.2013.00192 PMC3708450 23874338

[34] Godefroy E Manches O Dréno B Hochman T Rolnitzky L Labarrière N. Matrix metalloproteinase-2 conditions human dendritic cells to prime inflammatory T(H)2 cells via an IL-12- and OX40L-dependent pathway. Cancer Cell (2011) 19(3):333–46. doi: 10.1016/j.ccr.2011.01.037 PMC3073826 21397857

[35] Spranger S Bao R Gajewski TF. Melanoma-intrinsic β-catenin signalling prevents anti-tumour immunity. Nature (2015) 523(7559):231–5. doi: 10.1038/nature14404 25970248

[36] Holtzhausen A Zhao F Evans KS Tsutsui M Orabona C Tyler DS. Melanoma-derived Wnt5a promotes local dendritic-cell expression of IDO and immunotolerance: opportunities for pharmacologic enhancement of immunotherapy. Cancer Immunol Res (2015) 3(9):1082–95. doi: 10.1158/2326-6066.CIR-14-0167 PMC4927300 26041736

[37] Gubin MM Zhang X Schuster H Caron E Ward JP Noguchi T. Checkpoint blockade cancer immunotherapy targets tumour-specific mutant antigens. Nature (2014) 515(7528):577–81. doi: 10.1038/nature13988 PMC4279952 25428507

[38] Bodmer JL Schneider P Tschopp J. The molecular architecture of the TNF superfamily. Trends Biochem Sci (2002) 27(1):19–26. doi: 10.1016/s0968-0004(01)01995-8 11796220

[39] Wilson NS Dixit V Ashkenazi A. Death receptor signal transducers: nodes of coordination in immune signaling networks. Nat Immunol (2009) 10(4):348–55. doi: 10.1038/ni.1714 19295631

[40] Webster JD Vucic D. The balance of TNF mediated pathways regulates inflammatory cell death signaling in healthy and diseased tissues. Front Cell Dev Biol (2020) 21:365. doi: 10.3389/fcell.2020.00365 PMC7326080 32671059

[41] Tuomela K Ambrose AR Davis DM. Escaping death: how cancer cells and infected cells resist cell-mediated cytotoxicity. Front Immunol (2022) 13:867098. doi: 10.3389/fimmu.2022.867098 35401556 PMC8984481

[42] Rudd-Schmidt JA Hodel AW Noori T Lopez JA Cho HJ Verschoor S. Lipid order and charge protect killer T cells from accidental death. Nat Commun (2019) 10(1):5396. doi: 10.1038/s41467-019-13385-x 31776337 PMC6881447

[43] Wang X Liu M Geng N Du Y Li Z Gao X. Staphylococcus aureus mediates pyroptosis in bovine mammary epithelial cell via activation of NLRP3 inflammasome. Vet Res (2022) 53(1):10–0. doi: 10.1186/s13567-022-01027-y PMC8817610 35123552

[44] Singh R Letai A Sarosiek K. Regulation of apoptosis in health and disease: the balancing act of BCL-2 family proteins. Nat Rev Mol Cell Biol (2019) 20(3):175–93. doi: 10.1038/s41580-018-0089-8 PMC7325303 30655609

[45] Fei L Ren X Yu H Zhan Y. Targeting the CCL2/CCR2 axis in cancer immunotherapy: one stone, three birds? Front Immunol (2021) 3:771210. doi: 10.3389/fimmu.2021.771210 PMC8596464 34804061

[46] Lippitz BE. Cytokine patterns in patients with cancer: a systematic review. Lancet Oncol (2013) 14:e218–28. doi: 10.1016/S1470-2045(12)70582-X 23639322

[47] Seidel JA Otsuka A Kabashima K. Anti-PD-1 and anti-CTLA-4 therapies in cancer: mechanisms of action, efficacy, and limitations. Front Oncol (2018) 28:86. doi: 10.3389/fonc.2018.00086 PMC5883082 29644214

[48] Kelly PN Strasser A. The role of bcl-2 and its pro-survival relatives in tumourigenesis and cancer therapy. Cell Death Differ (2011) 18(9):1414–24. doi: 10.1038/cdd.2011.17 PMC3149740 21415859

[49] Saini KS Loi S de Azambuja E Metzger-Filho O Saini. ML Ignatiadis M. Targeting the PI3K/AKT/mTOR and Raf/MEK/ERK pathways in the treatment of breast cancer. Cancer Treat Rev (2013) 39(8):935–46. doi: 10.1016/j.ctrv.2013.03.009 23643661

[50] Schechter AL Stern DF Vaidyanathan L Decker SJ Drebin JA Greene MI. The neu oncogene: an erb-b-related gene encoding a 185,000-Mr tumour antigen. Nature (1984) 312(5994):513–6. doi: 10.1038/312513a0 6095109

[51] Hobbs GA Der CJ Rossman KL. RAS isoforms and mutations in cancer at a glance. J Cell Sci (2016) 129(7):1287–92. doi: 10.1242/jcs.182873 PMC4869631 26985062

[52] Luo J Manning BD Cantley LC. Targeting the PI3K-akt pathway in human cancer: rationale and promise. Cancer Cell (2003) 4(4):257–62. doi: 10.1016/s1535-6108(03)00248-4 14585353

[53] Ligresti G Militello L Steelman LS Cavallaro A Basile F Nicoletti F. PIK3CA mutations in human solid tumors: role in sensitivity to various therapeutic approaches. Cell Cycle (2009) 8(9):1352–8. doi: 10.4161/cc.8.9.8255 PMC3781181 19305151

[54] Davies H Bignell GR Cox C Stephens P Edkins S Clegg S. Mutations of the BRAF gene in human cancer. Nature (2002) 417(6892):949–54. doi: 10.1038/nature00766 12068308

[55] Garnett MJ Marais R. Guilty as charged: b-RAF is a human oncogene. Cancer Cell (2004) 6(4):313–9. doi: 10.1016/j.ccr.2004.09.022 15488754

[56] French AJ Sargent DJ Burgart LJ Foster NR Kabat BF Goldberg R. Prognostic significance of defective mismatch repair and BRAF V600E in patients with colon cancer. Clin Cancer Res (2008) 14(11):3408–15. doi: 10.1158/1078-0432.CCR-07-1489 PMC2674786 18519771

[57] Rubin BP Heinrich MC Corless CL. Gastrointestinal stromal tumour. Lancet (2007) 369(9574):1731–41. doi: 10.1016/S0140-6736(07)60780-6 17512858

[58] Thomas SJ Snowden JA Zeidler MP Danson SJ. The role of JAK/STAT signalling in the pathogenesis, prognosis and treatment of solid tumours. Br J Cancer (2015) 113:365–71. doi: 10.1038/bjc.2015.233 PMC4522639 26151455

[59] Lee SM. Is EGFR expression important in non-small cell lung cancer? Thorax (2006) 61:98–9. doi: 10.1136/thx.2005.047936 PMC2104566 16443704

[60] Janani B Vijayakumar M Priya K Kim JH Prabakaran DS Shahid M. EGFR-based targeted therapy for colorectal cancer-promises and challenges. Vaccines (Basel) (2022) 10(4):499. doi: 10.3390/vaccines10040499 35455247 PMC9030067

[61] Giancotti FG. Deregulation of cell signaling in cancer. FEBS Lett (2014) 19:2558–70. doi: 10.1016/j.febslet.2014.02.005 PMC4111980 24561200

[62] Flaherty KT Infante JR Daud A Gonzalez R Kefford RF Sosman J. Combined BRAF and MEK inhibition in melanoma with BRAF V600 mutations. N Engl J Med (2012) 367:1694–703. doi: 10.1056/NEJMoa1210093 PMC3549295 23020132

[63] Available at: https://www.fda.gov/drugs/resources-information-approved-drugs/fda-grants-accelerated-approval-adagrasib-kras-g12c-mutated-nsclc.

[64] Available at: https://www.fda.gov/drugs/resources-information-approved-drugs/fda-grants-accelerated-approval-sotorasib-kras-g12c-mutated-nsclc.

[65] Kim K-H Kim J-O Park J-Y Seo M-D Park SG. Antibody-drug conjugate targeting c-kit for the treatment of small cell lung cancer. Int J Mol Sci (2022) 23:2264. doi: 10.3390/ijms23042264 35216379 PMC8875948

[66] Nagano T Tachihara M Nishimura Y. Mechanism of resistance to epidermal growth factor receptor-tyrosine kinase inhibitors and a potential treatment strategy. Cells (2018) 7(11):212. doi: 10.3390/cells7110212 30445769 PMC6262543

[67] Liu G-H Chen T Zhang X Ma X-L Shi H-S. Small molecule inhibitors targeting the cancers. MedComm (2022) 3:e181. doi: 10.1002/mco2.181 36254250 PMC9560750

[68] Yang L Li A Lei Q Zhang Y. Tumor-intrinsic signaling pathways: key roles in the regulation of the immunosuppressive tumor microenvironment. J Hematol Oncol (2019) 12:125. doi: 10.1186/s13045-019-0804-8 31775797 PMC6880373

[69] Andreesen R Scheibenbogen C Brugger W Krause S Meerpohl HG Leser HG. Adoptive transfer of tumor cytotoxic macrophages generated *in vitro* from circulating blood monocytes: a new approach to cancer immunotherapy. Cancer Res (1990) 50(23):7450–6. 1701343

[70] Setlai BP Hull R Bida M Durandt C Mulaudzi TV Chatziioannou A. Immunosuppressive signaling pathways as targeted cancer therapies. Biomedicines (2022) 10(3):682. doi: 10.3390/biomedicines10030682 35327484 PMC8945019

[71] Lahmar Q Keirsse J Laoui D Movahedi K Van Overmeire E Van Ginderachter JA. Tissue-resident versus monocyte-derived macrophages in the tumor microenvironment. Biochim Biophys Acta (2016) 1865(1):23–34. doi: 10.1016/j.bbcan.2015.06.009 26145884

[72] Gordon S Taylor PR. Monocyte and macrophage heterogeneity. Nat Rev Immunol (2005) 5(12):953–64. doi: 10.1038/nri1733 16322748

[73] Brenner D Blaser H Mak TW. Regulation of tumour necrosis factor signalling: live or let die. Nat Rev Immunol (2015) 15:362–74. doi: 10.1038/nri3834 26008591

[74] Zins K Abraham D Sioud M Aharinejad S. Colon cancer cell-derived tumor necrosis factor-alpha mediates the tumor growth-promoting response in macrophages by up-regulating the colony-stimulating factor-1 pathway. Cancer Res (2007) 67:1038–45. doi: 10.1158/0008-5472.CAN-06-2295 17283136

[75] Tuzlak S Dejean AS Iannacone M Quintana FJ Waisman A Ginhoux F. Repositioning TH cell polarization from single cytokines to complex help. Nat Immunol (2021) 22(10):1210–7. doi: 10.1038/s41590-021-01009-w 34545250

[76] Tauriello DV Calon A Lonardo E Batlle E. Determinants of metastatic competency in colorectal cancer. Mol Oncol (2017) 11:97–119. doi: 10.1002/1878-0261.12018 28085225 PMC5423222

[77] Kessel A Haj T Peri R Snir A Melamed D Sabo E. Human CD19(+)CD25(high) b regulatory cells suppress proliferation of CD4(+) T cells and enhance Foxp3 and CTLA-4 expression in T-regulatory cells. Autoimmun Rev (2012) 11(9):670–7. doi: 10.1016/j.autrev.2011.11.018 22155204

[78] Mantovani A Marchesi F Malesci A Laghi L Allavena P. Tumour-associated macrophages as treatment targets in oncology. Nat Rev Clin Oncol (2017) 14(7):399–416. doi: 10.1038/nrclinonc.2016.217 28117416 PMC5480600

[79] Mao X Xu J Wang W Liang C Hua J Liu J. Crosstalk between cancer-associated fibroblasts and immune cells in the tumor microenvironment: new findings and future perspectives. Mol Cancer (2021) 20(1):131. doi: 10.1186/s12943-021-01428-1 34635121 PMC8504100

[80] Tian L Lei A Tan T Zhu M Zhang L Mou H. Macrophage-based combination therapies as a new strategy for cancer immunotherapy. Kidney Dis (Basel) (2021) 8(1):26–43. doi: 10.1159/000518664 35224005 PMC8820174

[81] Duan Q Zhang H Zheng J Zhang L. Turning cold into hot: firing up the tumor microenvironment. Trends Cancer (2020) 6(7):605–18. doi: 10.1016/j.trecan.2020.02.022 32610070

[82] Kaneda MM Messer KS Ralainirina N Li H Leem CJ Gorjestani S. PI3Kγ is a molecular switch that controls immune suppression. Nature (2016) 539(7629):437–42. doi: 10.1038/nature19834 PMC5479689 27642729

[83] Krishnamoorthy M Gerhardt L Maleki Vareki S. Immunosuppressive effects of myeloid-derived suppressor cells in cancer and immunotherapy. Cells (2021) 10(5):1170. doi: 10.3390/cells10051170 34065010 PMC8150533

[84] Zhang L Tian L Dai X Yu H Wang J Lei A. Pluripotent stem cell-derived CAR-macrophage cells with antigen-dependent anti-cancer cell functions. J Hematol Oncol (2020) 13(1):153. doi: 10.1186/s13045-020-00983-2 33176869 PMC7656711

[85] Saini KS Svane IM Juan M Barlesi F André F. Manufacture of adoptive cell therapies at academic cancer centers: scientific, safety and regulatory challenges. Ann Oncol (2022) 33(1):6–12. doi: 10.1016/j.annonc.2021.09.020 34655734

[86] Zhang H Passang T Ravindranathan S Bommireddy R Jajja MR Yang L. The magic of small-molecule drugs during ex vivo expansion in adoptive cell therapy. Front Immunol (2023) 14:1154566. doi: 10.3389/fimmu.2023.1154566 37153607 PMC10160370

[87] Chellappa S Kushekhar K Munthe LA Tjønnfjord GE Aandahl EM Okkenhaug K. The PI3K p110d isoform inhibitor idelalisib preferentially inhibits human regulatory T cell function. J Immunol (2019) 202:1397–405. doi: 10.4049/jimmunol.1701703 30692213

[88] Funk CR Wang S Chen KZ Waller A Sharma A Edgar CL. PI3Kd/g inhibition promotes human CART cell epigenetic and metabolic reprogramming to enhance antitumor cytotoxicity. Blood (2022) 139:523–37. doi: 10.1182/blood.2021011597 PMC8796652 35084470

[89] Zhang H Hu Y Shao M Teng X Jiang P Wang X. Dasatinib enhances anti-leukemia efficacy of chimeric antigen receptor T cells by inhibiting cell differentiation and exhaustion. J Hematol Oncol (2021) 14:113. doi: 10.1186/s13045-021-01117-y 34289897 PMC8293573

[90] Beltra JC Manne S Abdel-Hakeem MS Kurachi M Giles JR Chen Z. Developmental relationships of four exhausted CD8(+) T cell subsets reveals underlying transcriptional and epigenetic landscape control mechanisms. Immunity (2020) 52:825–841.e828. doi: 10.1016/j.immuni.2020.04.014 32396847 PMC8360766

[91] Wang Y Tong C Dai H Wu Z Han X Guo Y. Low-dose decitabine priming endows CAR T cells with enhanced and persistent antitumour potential via epigenetic reprogramming. Nat Commun (2021) 12:409. doi: 10.1038/s41467-020-20696-x 33462245 PMC7814040

[92] Kagoya Y Nakatsugawa M Yamashita Y Ochi T Guo T Anczurowski M. BET bromodomain inhibition enhances T cell persistence and function in adoptive immunotherapy models. J Clin Invest (2016) 126:3479–94. doi: 10.1172/JCI86437 PMC5004946 27548527

[93] Lemoine J Morin F Di Blasi R Vercellino L Cuffel A Benlachgar N. Lenalidomide exposure at time of CAR T-cells expansion enhances response of Refractory/Relapsed aggressive Large b-cell lymphomas. Blood (2021) 138:1433–3. doi: 10.1182/blood-2021-151109

[94] Ali SA Shi V Maric I Wang M Stroncek DF Rose JJ. T Cells expressing an anti-b-cell maturation antigen chimeric antigen receptor cause remissions of multiple myeloma. Blood (2016) 128:1688–700. doi: 10.1182/blood-2016-04-711903 PMC5043125 27412889

[95] Hupperetz C Lah S Kim H Kim CH. CAR T cell immunotherapy beyond haematological malignancy. Immune Netw (2022) 22(1):e6. doi: 10.4110/in.2022.22.e6 35291659 PMC8901698

[96] Safarzadeh Kozani P Safarzadeh Kozani P Ahmadi Najafabadi M Yousefi F Mirarefin SMJ Rahbarizadeh F. Recent advances in solid tumor CAR-T cell therapy: driving tumor cells from hero to zero? Front Immunol (2022) 11:795164. doi: 10.3389/fimmu.2022.795164 PMC9130586 35634281

[97] Gabrilovich DI Nagaraj S. Myeloid-derived suppressor cells as regulators of the immune system. Nat Rev Immunol (2009) 9:162–74. doi: 10.1038/nri2506 PMC2828349 19197294

[98] Xia J Minamino S Kuwabara K. CAR-expressing NK cells for cancer therapy: a new hope. Biosci Trends (2020) 14:354–9. doi: 10.5582/bst.2020.03308 32893255

[99] Klingemann H. Are natural killer cells superior CAR drivers? Oncoimmunology (2014) 3:e28147. doi: 10.4161/onci.28147 25340009 PMC4203506

[100] Besser MJ Shapira-Frommer R Itzhaki O Treves AJ Zippel DB Levy D. Adoptive transfer of tumor-infiltrating lymphocytes in patients with metastatic melanoma: intent-to-treat analysis and efficacy after failure to prior immunotherapies. Clin Cancer Res (2013) 19:4792–800. doi: 10.1158/1078-0432.CCR-13-0380 23690483

[101] Stevanović S Draper LM Langhan MM Campbell TE Kwong ML Wunderlich JR. Complete regression of metastatic cervical cancer after treatment with human papillomavirus-targeted tumor-infiltrating T cells. J Clin Oncol Off J Am Soc Clin Oncol (2015) 33:1543–50. doi: 10.1200/JCO.2014.58.9093 PMC4417725 25823737

[102] Zacharakis N Chinnasamy H Black M Xu H Lu Y-C Zheng Z. Immune recognition of somatic mutations leading to complete durable regression in metastatic breast cancer. Nat Med (2018) 24:724–30. doi: 10.1038/s41591-018-0040-8 PMC6348479 29867227

[103] Baulu E Gardet C Chuvin N Depil S. TCR-engineered T cell therapy in solid tumors: state of the art and perspectives. Sci Adv (2023) 9(7):eadf3700. doi: 10.1126/sciadv.adf3700 36791198 PMC9931212

[104] Tsimberidou AM Van Morris K Vo HH Eck S Lin YF Rivas JM. T-Cell receptor-based therapy: an innovative therapeutic approach for solid tumors. J Hematol Oncol (2021) 14(1):102. doi: 10.1186/s13045-021-01115-0 34193217 PMC8243554

[105] Depil S Duchateau P Grupp SA Mufti G Poirot L. 'Off-the-shelf' allogeneic CAR T cells: development and challenges. Nat Rev Drug Discovery (2020) 19(3):185–99. doi: 10.1038/s41573-019-0051-2 31900462

[106] Aftab BT Sasu B Krishnamurthy J Gschweng E Alcazer V Depil S. Toward “off-the-shelf” allogeneic CAR T cells. Adv Cell Gene Ther (2020) 3:e86. doi: 10.1002/acg2.86

[107] Mullard A. FDA Approval of immunocore's first-in-class TCR therapeutic broadens depth of the T cell engager platform. Nat Rev Drug Discovery (2022) 21(3):170. doi: 10.1038/d41573-022-00031-3 35145259

[108] Zhou S Liu M Ren F Meng X Yu J. The landscape of bispecific T cell engager in cancer treatment. biomark Res (2021) 9:38. doi: 10.1186/s40364-021-00294-9 34039409 PMC8157659

[109] Muthuswamy R Wang L Pitteroff J Gingrich JR Kalinski P. Combination of IFNalpha and poly-I:C reprograms bladder cancer microenvironment for enhanced CTL attraction. J Immunother Cancer (2015) 3:6. doi: 10.1186/s40425-015-0050-8 25806105 PMC4371844

[110] Watts TH. TNF/TNFR family members in costimulation of T cell responses. Annu Rev Immunol (2005) 23:23–68. doi: 10.1146/annurev.immunol.23.021704.115839 15771565

[111] Janakiram M Shah UA Liu W Zhao A Schoenberg MP Zang X. The third group of the B7-CD28 immune checkpoint family: HHLA2, TMIGD2, B7x, and B7-H3. Immunol Rev (2017) 276:26–39. doi: 10.1111/imr.12521 28258693 PMC5338461

[112] Marcucci F Rumio C. Depleting tumor cells expressing immune checkpoint ligands-a new approach to combat cancer. Cells (2021) 10(4):872. doi: 10.3390/cells10040872 33921301 PMC8069236

[113] Taube JM Anders RA Young GD Xu H Sharma R McMiller TL. Colocalization of inflammatory response with B7-h1 expression in human melanocytic lesions supports an adaptive resistance mechanism of immune escape. Sci Transl Med (2012) 4(127):127ra37. doi: 10.1126/scitranslmed.3003689 PMC3568523 22461641

[114] Lupo KB Matosevic S. Natural killer cells as allogeneic effectors in adoptive cancer immunotherapy. Cancers (Basel) (2019) 11(6):769. doi: 10.3390/cancers11060769 31163679 PMC6628161

[115] Marin-Acevedo JA Kimbrough EO Lou Y. Next generation of immune checkpoint inhibitors and beyond. J Hematol Oncol (2021) 14:45. doi: 10.1186/s13045-021-01056-8 33741032 PMC7977302

[116] He Y Cao J Zhao C Li X Zhou C Hirsch FR. TIM-3, a promising target for cancer immunotherapy. Oncol Targets Ther (2018) 11:7005–9. doi: 10.2147/OTT.S170385 PMC6198883 30410357

[117] Anderson AC Joller N Kuchroo VK. Lag-3, Tim-3, and TIGIT: co-inhibitory receptors with specialized functions in immune regulation. Immunity (2016) 44(5):989–1004. doi: 10.1016/j.immuni.2016.05.001 27192565 PMC4942846

[118] Zhou Q Munger ME Veenstra RG Weigel BJ Hirashima M Munn DH. Coexpression of Tim-3 and PD-1 identifies a CD8+ T-cell exhaustion phenotype in mice with disseminated acute myelogenous leukemia. Blood (2011) 117(17):4501–10. doi: 10.1182/blood-2010-10-310425 PMC3099570 21385853

[119] Sakuishi K Ngiow SF Sullivan JM Teng MW Kuchroo VK Smyth MJ. TIM3+FOXP3+ regulatory T cells are tissue-specific promoters of T-cell dysfunction in cancer. Oncoimmunology (2013) 2(4):e23849. doi: 10.4161/onci.23849 23734331 PMC3654601

[120] Du W Yang M Turner A Xu C Ferris RL Huang J. TIM-3 as a target for cancer immunotherapy and mechanisms of action. Int J Mol Sci (2017) 18(3):645. doi: 10.3390/ijms18030645 28300768 PMC5372657

[121] Ruffo E Wu RC Bruno TC Workman CJ Vignali DAA. Lymphocyte-activation gene 3 (LAG3): the next immune checkpoint receptor. Semin Immunol (2019) 42:101305. doi: 10.1016/j.smim.2019.101305 31604537 PMC6920665

[122] Marin-Acevedo JA Dholaria B Soyano AE Knutson KL Chumsri S Lou Y. Next generation of immune checkpoint therapy in cancer: new developments and challenges. J Hematol Oncol (2018) 11(1):39. doi: 10.1186/s13045-018-0582-8 29544515 PMC5856308

[123] Graydon CG Mohideen S Fowke KR. LAG3's enigmatic mechanism of action. Front Immunol (2020) 11:615317. doi: 10.3389/fimmu.2020.615317 33488626 PMC7820757

[124] Goldberg MV Drake CG. LAG-3 in cancer immunotherapy. Curr Top Microbiol Immunol (2011) 344:269–78. doi: 10.1007/82_2010_114 PMC4696019 21086108

[125] Shi AP Tang XY Xiong YL Zheng KF Liu YJ Shi XG. Immune checkpoint LAG3 and its ligand FGL1 in cancer. Front Immunol (2022) 17:785091. doi: 10.3389/fimmu.2021.785091 PMC8801495 35111155

[126] Andrews LP Marciscano AE Drake CG Vignali DAA. LAG3 (CD223) as a cancer immunotherapy target. Immunol Rev (2017) 276(1):80–96. doi: 10.1111/imr.12519 28258692 PMC5338468

[127] Tawbi HA Schadendorf D Lipson EJ Ascierto PA Matamala L Castillo Gutiérrez E. RELATIVITY-047 investigators. relatlimab and nivolumab versus nivolumab in untreated advanced melanoma. N Engl J Med (2022) 386(1):24–34. doi: 10.1056/NEJMoa2109970 34986285 PMC9844513

[128] Chauvin JM Zarour HM. TIGIT in cancer immunotherapy. J Immunother Cancer (2020) 8:e000957. doi: 10.1136/jitc-2020-000957 32900861 PMC7477968

[129] Sun Y Luo J Chen Y Cui J Lei Y Cui Y. Combined evaluation of the expression status of CD155 and TIGIT plays an important role in the prognosis of LUAD (lung adenocarcinoma). Int Immunopharmacol (2020) 80:106198. doi: 10.1016/j.intimp.2020.106198 31954274

[130] Ostroumov D Duong S Wingerath J Woller N Manns MP Timrott K. Transcriptome profiling identifies TIGIT as a marker of T-cell exhaustion in liver cancer. Hepatology (2021) 73(4):1399–418. doi: 10.1002/hep.31466 32716559

[131] Cho BC Abreu DR Hussein M Cobo M Patel AJ Secen N. Tiragolumab plus atezolizumab versus placebo plus atezolizumab as a first-line treatment for PD-L1-selected non-small-cell lung cancer (CITYSCAPE): primary and follow-up analyses of a randomised, double-blind, phase 2 study. Lancet Oncol (2022) 23(6):781–92. doi: 10.1016/S1470-2045(22)00226-1 35576957

[132] Roche News release. Roche reports interim results for phase III SKYSCRAPER-01 study in PD-L1-high metastatic non-small cell lung cancer . Available at: https://bit.ly/37EbDJX (Accessed May 12, 2022).

[133] Phan GQ Yang JC Sherry RM Hwu P Topalian SL Schwartzentruber DJ. Cancer regression and autoimmunity induced by cytotoxic t lymphocyte-associated antigen 4 blockade in patients with metastatic melanoma. Proc Natl Acad Sci U.S.A. (2003) 100(14):8372–7. doi: 10.1073/pnas.1533209100 PMC166236 12826605

[134] Rowshanravan B Halliday N Sansom DM. CTLA-4: a moving target in immunotherapy. Blood (2018) 131:58–67. doi: 10.1182/blood-2017-06-741033 29118008 PMC6317697

[135] Hu P Liu Q Deng G Zhang J Liang N Xie J. The prognostic value of cytotoxic T-lymphocyte antigen 4 in cancers: a systematic review and meta-analysis. Sci Rep (2017) 7:42913. doi: 10.1038/srep42913 28211499 PMC5314410

[136] Ishida Y Agata Y Shibahara K Honjo T. Induced expression of PD-1, a novel member of the immunoglobulin gene superfamily, upon programmed cell death. EMBO J (1992) 11:3887–95. doi: 10.1002/j.1460-2075.1992.tb05481.x PMC556898 1396582

[137] Zak KM Grudnik P Magiera K Dömling A Dubin G Holak TA. Structural biology of the immune checkpoint receptor PD-1 and its ligands PD-L1/PD-L2. Structure (2017) 25:1163–74. doi: 10.1016/j.str.2017.06.011 28768162

[138] Dong H Zhu G Tamada K Chen L. B7-H1, a third member of the B7 family, co-stimulates T-cell proliferation and interleukin-10 secretion. Nat Med (1999) 5:1365–9. doi: 10.1038/70932 10581077

[139] Han Y Liu D Li L. PD-1/PD-L1 pathway: current researches in cancer. Am J Cancer Res (2020) 10:727. 32266087 PMC7136921

[140] Herbst RS Giaccone G De Marinis F Reinmuth N Vergnenegre A Barrios CH. Atezolizumab for first-line treatment of PD-L1–selected patients with NSCLC. N Engl J Med (2020) 383:1328–39. doi: 10.1056/NEJMoa1917346 32997907

[141] Stewart R Morrow M Hammond SA Mulgrew K Marcus D Poon E. Identification and characterization of MEDI4736, an antagonistic anti-PD-L1 monoclonal antibody. Cancer Immunol Res (2015) 3:1052–62. doi: 10.1158/2326-6066.CIR-14-0191 25943534

[142] Boyerinas B Jochems C Fantini M Heery CR Gulley JL Tsang KY. Antibody-dependent cellular cytotoxicity activity of a novel anti-PD-L1 antibody avelumab (MSB0010718C) on human tumor cells. Cancer Immunol Res (2015) 3:1148–57. doi: 10.1158/2326-6066.CIR-15-0059 PMC4739754 26014098

[143] Brahmer JR Tykodi SS Chow LQ Hwu WJ Topalian SL Hwu P. Safety and activity of anti-PD-L1 antibody in patients with advanced cancer. N Engl J Med (2012) 366:2455–65. doi: 10.1056/NEJMoa1200694 PMC3563263 22658128

[144] Shiravand Y Khodadadi F Kashani SMA Hosseini-Fard SR Hosseini S Sadeghirad H. Immune checkpoint inhibitors in cancer therapy. Curr Oncol (2022) 29:3044–60. doi: 10.3390/curroncol29050247 PMC9139602 35621637

[145] Wang S Xie K Liu T. Cancer immunotherapies: from efficacy to resistance mechanisms - not only checkpoint matters. Front Immunol (2021) 21:690112. doi: 10.3389/fimmu.2021.690112 PMC8335396 34367148

[146] Gandhi S Opyrchal M Grimm M Slomba R Kokolus KM Battaglia S. (2022). Abstract CT145: systemic rintatolimod and interferon-α2b selectively reprogram local tumor microenvironment in patients with metastatic triple negative breast cancer for enhanced influx of cytotoxic T-lymphocytes but not regulatory T-cells. Cancer Res (2022) 82(Suppl 12):CT145. doi: 10.1158/1538-7445.AM2022-CT14510.1158/1538-7445.AM2022-CT145

[147] Diamond MS Kinder M Matsushita H Mashayekhi M Dunn GP Archambault JM. Type I interferon is selectively required by dendritic cells for immune rejection of tumors. J Exp Med (2011) 208:1989–2003. doi: 10.1084/jem.20101158 21930769 PMC3182061

[148] Müller E Speth M Christopoulos PF Lunde A Avdagic A Øynebråten I. Both type I and type II interferons can activate antitumor M1 macrophages when combined with TLR stimulation. Front Immunol (2018) 9:2520. doi: 10.3389/fimmu.2018.02520 30450098 PMC6224375

[149] Sistigu A Yamazaki T Vacchelli E Chaba K Enot DP Adam J. Cancer cell–autonomous contribution of type I interferon signaling to the efficacy of chemotherapy. Nat Med (2014) 20:1301–9. doi: 10.1038/nm.3708 25344738

[150] Di S Zhou M Pan Z Sun R Chen M Jiang H. Combined adjuvant of poly I:C improves antitumor effects of CAR-T cells. Front Oncol (2019) 9:241. doi: 10.3389/fonc.2019.00241 31058074 PMC6481273

[151] Wherry EJ Kurachi M. Molecular and cellular insights into T cell exhaustion. Nat Rev Immunol (2015) 15(8):486–99. doi: 10.1038/nri3862 PMC4889009 26205583

[152] Huang KCY Chiang SF Chang HY Chen WT Yang PC Chen TW. Engineered sTRAIL-armed MSCs overcome STING deficiency to enhance the therapeutic efficacy of radiotherapy for immune checkpoint blockade. Cell Death Dis (2022) 13:610. doi: 10.1038/s41419-022-05069-0 35835756 PMC9283452

[153] Deng L Liang H Xu M Yang X Burnette B Arina A. STING-dependent cytosolic DNA sensing promotes radiation-induced type I interferon-dependent antitumor immunity in immunogenic tumors. Immunity (2014) 41:843–52. doi: 10.1016/j.immuni.2014.10.019 PMC5155593 25517616

[154] Xia T Konno H Ahn J Barber GN. Deregulation of STING signaling in colorectal carcinoma constrains DNA damage responses and correlates with tumorigenesis. Cell Rep (2016) 14:282–97. doi: 10.1016/j.celrep.2015.12.029 PMC4845097 26748708

[155] Corrales L Glickman LH McWhirter SM Kanne DB Sivick KE Katibah GE. Direct activation of STING in the tumor microenvironment leads to potent and systemic tumor regression and immunity. Cell Rep (2015) 11:1018–30. doi: 10.1016/j.celrep.2015.04.031 PMC4440852 25959818

[156] Olivier H James KL. Inhibition of MHC II gene transcription by nitric oxide and antioxidants. Curr Pharm Des (2004) 10:893–8. doi: 10.2174/1381612043452893 PMC2633593 15032692

[157] Gabrilovich D Ostrand-Rosenberg S Bronte V. Coordinated regulation of myeloid cells by tumours. Nat Rev Immunol (2012) 12:253–68. doi: 10.1038/nri3175 PMC3587148 22437938

[158] Xiong H Zhu C Li F Hegazi R He K Babyatsky M. Inhibition of interleukin-12 p40 transcription and NF-kappaB activation by nitric oxide in murine macrophages and dendritic cells. J Biol Chem (2004) 279:10776–83. doi: 10.1074/jbc.M313416200 14679201

[159] Grzywa TM Sosnowska A Matryba P Rydzynska Z Jasinski M Nowis D. Myeloid cell-derived arginase in cancer immune response. Front Immunol (2020) 11:938. doi: 10.3389/fimmu.2020.00938 32499785 PMC7242730

[160] Rodriguez PC Quiceno DG Zabaleta J Ortiz B Zea AH Piazuelo MB. Arginase I production in the tumor microenvironment by mature myeloid cells inhibits T-cell receptor expression and antigen-specific T-cell responses. Cancer Res (2004) 64:5839–49. doi: 10.1158/0008-5472.CAN-04-0465 15313928

[161] Alexandrou C Al-Aqbi SS Higgins JA Boyle W Karmokar A Andreadi C. Sensitivity of colorectal cancer to arginine deprivation therapy is shaped by differential expression of urea cycle enzymes. Sci Rep (2018) 8(1):12096. doi: 10.1038/s41598-018-30591-7 30108309 PMC6092409

[162] Zea AH Rodriguez PC Culotta KS Hernandez CP DeSalvo J Ochoa JB. Arginine modulates CD3ζ expression and T cell function in activated human T lymphocytes. Cell Immunol (2004) 232:21–31. doi: 10.1016/j.cellimm.2005.01.004 15922712

[163] Mondanelli G Bianchi R Pallotta MT Orabona C Albini E Iacono A. A relay pathway between arginine and tryptophan metabolism confers immunosuppressive properties on dendritic cells. Immunity (2017) 46:233–44. doi: 10.1016/j.immuni.2017.01.005 PMC5337620 28214225

[164] Steggerda SM Bennett MK Chen J Emberley E Huang T Janes JR. Inhibition of arginase by CB-1158 blocks myeloid cell-mediated immune suppression in the tumor microenvironment. J Immunother Cancer (2017) 5:101. doi: 10.1186/s40425-017-0308-4 29254508 PMC5735564

[165] Vigano S Alatzoglou D Irving M Ménétrier-Caux C Caux C Romero P. Targeting adenosine in cancer immunotherapy to enhance T-cell function. Front Immunol (2019) 10:925. doi: 10.3389/fimmu.2019.00925 31244820 PMC6562565

[166] Pasquini S Contri C Borea PA Vincenzi F Varani K. Adenosine and inflammation: here, there and everywhere. Int J Mol Sci (2021) 22:7685. doi: 10.3390/ijms22147685 34299305 PMC8304851

[167] Beavis PA Stagg J Darcy PK Smyth MJ. CD73: a potent suppressor of antitumor immune responses. Trends Immunol (2012) 33:231–7. doi: 10.1016/j.it.2012.02.009 22487321

[168] Mandapathil M Hilldorfer B Szczepanski MJ Czystowska M Szajnik M Ren J. Generation and accumulation of immunosuppressive adenosine by human CD4+CD25highFOXP3+ regulatory T cells. J Biol Chem (2010) 285:7176–86. doi: 10.1074/jbc.M109.047423 PMC2844167 19858205

[169] Hoskin DW Mader JS Furlong SJ Conrad DM Blay J. Inhibition of T cell and natural killer cell function by adenosine and its contribution to immune evasion by tumor cells (Review). Int J Oncol (2008) 32:527–35. doi: 10.3892/ijo.32.3.527 18292929

[170] Vitale I Manic G Coussens LM Kroemer G Galluzzi L. Macrophages and metabolism in the tumor microenvironment. Cell Metab (2019) 30(1):36–50. doi: 10.1016/j.cmet.2019.06.001 31269428

[171] Ryzhov SV Pickup MW Chytil A Gorska AE Zhang Q Owens P. Role of TGF-beta signaling in generation of CD39+CD73+ myeloid cells in tumors. J Immunol (2014) 193:3155–64. doi: 10.4049/jimmunol.1400578 PMC4157098 25127858

[172] Ferrante CJ Pinhal-Enfield G Elson G Cronstein BN Hasko G Outram S. The adenosine-dependent angiogenic switch of macrophages to an M2-like phenotype is independent of interleukin-4 receptor alpha (IL-4Rα) signaling. Inflammation (2013) 36(4):921–31. doi: 10.1007/s10753-013-9621-3 PMC3710311 23504259

[173] Bova V Filippone A Casili G Lanza M Campolo M Capra AP. Adenosine targeting as a new strategy to decrease glioblastoma aggressiveness. Cancers (2022) 14:4032. doi: 10.3390/cancers14164032 36011024 PMC9406358

[174] Gao ZG Jacobson KA. A2B adenosine receptor and cancer. Int J Mol Sci (2019) 20(20):5139. doi: 10.3390/ijms20205139 31627281 PMC6829478

[175] Fong L Hotson A Powderly JD Sznol M Heist RS Choueiri TK. Adenosine 2A receptor blockade as an immunotherapy for treatment-refractory renal cell cancer. Cancer Discovery (2020) 10:40–53. doi: 10.1158/2159-8290 31732494 PMC6954326

[176] Harshman LC Chu M George S Hughes BGM Carthon BC Fong L. Adenosine receptor blockade with ciforadenant +/- atezolizumab in advanced metastatic castration-resistant prostate cancer (mCRPC). J Clin Oncol (2020) 38(6_suppl):129–9. doi: 10.1200/jco.2020.38.6

[177] Bilir C Sarisozen C. Indoleamine 2,3-dioxygenase (IDO): only an enzyme or a checkpoint controller? J. Oncol Sci (2017) 3:52–6. doi: 10.1016/j.jons.2017.04.001

[178] Platten M Wick W Van den Eynde BJ. Tryptophan catabolism in cancer: beyond IDO and tryptophan depletion. Cancer Res (2012) 72:5435–40. doi: 10.1158/0008-5472.CAN-12-0569 23090118

[179] Munn DH Sharma MD Baban B Harding HP Zhang Y Ron D. GCN2 kinase in T cells mediates proliferative arrest and anergy induction in response to indoleamine 2,3-dioxygenase. Immunity (2005) 22:633–42. doi: 10.1016/j.immuni.2005.03.013 15894280

[180] Frumento G Rotondo R Tonetti M Damonte G Benatti U Ferrara GB. Tryptophan-derived catabolites are responsible for inhibition of T and natural killer cell proliferation induced by indoleamine 2,3-dioxygenase. J Exp Med (2002) 196:459–68. doi: 10.1084/jem.20020121 PMC2196046 12186838

[181] Della CM Carlomagno S Frumento G Balsamo M Cantoni C Conte R. The tryptophan catabolite l-kynurenine inhibits the surface expression of NKp46- and NKG2D-activating receptors and regulates NK-cell function. Blood (2006) 108:4118–25. doi: 10.1182/blood-2006-03-006700 16902152

[182] Vito A El-Sayes N Mossman K. Hypoxia-driven immune escape in the tumor microenvironment. Cells (2020) 9:992. doi: 10.3390/cells9040992 32316260 PMC7227025

[183] Mallikarjuna P Raviprakash TS Aripaka K Ljungberg B Landström M. Interactions between TGF-β type I receptor and hypoxia-inducible factor-α mediates a synergistic crosstalk leading to poor prognosis for patients with clear cell renal cell carcinoma. Cell Cycle (2019) 18(17):2141–56. doi: 10.1080/15384101.2019.1642069 PMC6986558 31339433

[184] Huang Y Chen Z Lu T Bi G Li M Liang J. HIF-1α switches the functionality of TGF-β signaling via changing the partners of smads to drive glucose metabolic reprogramming in non-small cell lung cancer. J Exp Clin Cancer Res (2021) 40:398. doi: 10.1186/s13046-021-02188-y 34930376 PMC8690885

[185] Clambey ET McNamee EN Westrich JA Glover LE Campbell EL Jedlicka P. Hypoxia-inducible factor-1 alpha-dependent induction of FoxP3 drives regulatory T-cell abundance and function during inflammatory hypoxia of the mucosa. Proc Natl Acad Sci USA (2012) 109(41):E2784–93. doi: 10.1073/pnas.1202366109 PMC3478644 22988108

[186] Lunt SY Vander Heiden MG. Aerobic glycolysis: meeting the metabolic requirements of cell proliferation. Annu Rev Cell Dev Bio (2011) 27:441–64. doi: 10.1146/annurev-cellbio-092910-154237 21985671

[187] Gottlob K Majewski N Kennedy S Kandel E Robey RB Hay N. Inhibition of early apoptotic events by Akt/PKB is dependent on the first committed step of glycolysis and mitochondrial hexokinase. Genes Dev (2001) 15(11):1406–18. doi: 10.1101/gad.889901 PMC312709 11390360

[188] Elstrom RL Bauer DE Buzzai M Karnauskas R Harris MH Plas DR. Akt stimulates aerobic glycolysis in cancer cells. Cancer Res (2004) 64(11):3892–9. doi: 10.1158/0008-5472.CAN-03-2904 15172999

[189] Tanaka A Sakaguchi S. Regulatory TTargeting treg cells in cancer immunotherapy. Cell Res (2017) 27(1):109–18. doi: 10.1038/cr.2016.151.1002/eji.201847659 PMC5223231 27995907

[190] Jonasch E Donskov F Iliopoulos O Rathmell K Narayan V Maughan BL. Phase II study of the oral HIF-2α inhibitor MK-6482 for Von hippel-lindau disease–associated renal cell carcinoma. J Clin Oncol (2020) 38:5003. doi: 10.1200/JCO.2020.38.15_suppl.5003

[191] Markovič T Jakopin Ž. Dolenc MS Mlinarič-Raščan I. Structural features of subtype-selective EP receptor modulators. Drug Discovery Today (2017) 22:57–71. doi: 10.1016/j.drudis.2016.08.003 27506873

[192] Kalinski P. Regulation of immune responses by prostaglandin E2. J Immunol (2012) 188:21–8. doi: 10.4049/jimmunol.1101029 PMC3249979 22187483

[193] Yokoi A Ochiya T. Exosomes and extracellular vesicles: rethinking the essential values in cancer biology. Semin Cancer Biol (2021) 74:79–91. doi: 10.1016/j.semcancer.2021.03.032 33798721

[194] Kowal J Tkach M Thery C. Biogenesis and secretion of exosomes. Curr Opin Cell Biol (2014) 29:116–25. doi: 10.1016/j.ceb.2014.05.004 24959705

[195] Sui H Liu H Yao M Su Y Qu P. MicroRNAs/LncRNAs modulate MDSCs in tumor microenvironment. Front Oncol (2022) 12:772351. doi: 10.3389/fonc.2022.772351 35359390 PMC8963964

[196] Li Y Zhao W Wang Y Wang H Liu S. Extracellular vesicle-mediated crosstalk between pancreatic cancer and stromal cells in the tumor microenvironment. J Nanobiotechnol (2022) 20(1):208. doi: 10.1186/s12951-022-01382-0 PMC9063273 35501802

[197] Mathieu M Martin-Jaular L Lavieu G Théry C. Specificities of secretion and uptake of exosomes and other extracellular vesicles for cell-to-cell communication. Nat Cell Biol (2019) 21(1):9–17. doi: 10.1038/s41556-018-0250-9 30602770

[198] Xiang X Poliakov A Liu C Liu Y Deng ZB Wang J. Induction of myeloid-derived suppressor cells by tumor exosomes. Int J Cancer (2009) 124(11):2621–33. doi: 10.1002/ijc.24249 PMC2757307 19235923

[199] Mathew M Zade M Mezghani N Patel R Wang Y Momen-Heravi F. Extracellular vesicles as biomarkers in cancer immunotherapy. Cancers (Basel) (2020) 12:2825. doi: 10.3390/cancers12102825 33007968 PMC7600903

[200] Théry C Ostrowski M Segura E. Membrane vesicles as conveyors of immune responses. Nat Rev Immunol (2009) 9(8):581–93. doi: 10.1038/nri2567 19498381

[201] Valadi H Ekström K Bossios A Sjöstrand M Lee JJ Lötvall JO. Exosome-mediated transfer of mRNAs and microRNAs is a novel mechanism of genetic exchange between cells. Nat Cell Biol (2007) 9(6):654–9. doi: 10.1038/ncb1596 17486113

[202] Wang J Zeng H Zhang H Han Y. The role of exosomal PD-L1 in tumor immunotherapy. Transl Oncol (2021) 14(5):101047. doi: 10.1016/j.tranon.2021.101047 33647542 PMC7921878

[203] de Miguel-Perez D Russo A Arrieta O Ak M Barron F Gunasekaran M. Extracellular vesicle PD-L1 dynamics predict durable response to immune-checkpoint inhibitors and survival in patients with non-small cell lung cancer. J Exp Clin Cancer Res (2022) 41:186. doi: 10.1186/s13046-022-02379-1 35650597 PMC9161571

[204] Takakura Y Matsumoto A Takahashi Y. Therapeutic application of small extracellular vesicles (sEVs): pharmaceutical and pharmacokinetic challenges. Biol Pharm Bull (2020) 43(4):576–83. doi: 10.1248/bpb.b19-00831 32238700

[205] Tuomisto AE Mäkinen MJ Väyrynen JP. Systemic inflammation in colorectal cancer: underlying factors, effects, and prognostic significance. World J Gastroenterol (2019) 25:4383–404. doi: 10.3748/wjg.v25.i31.4383 PMC6710177 31496619

[206] Yang Y Wang Y. Role of epigenetic regulation in plasticity of tumor immune microenvironment. Front Immunol (2021) 2:640369. doi: 10.3389/fimmu.2021.640369 PMC8051582 33868269

[207] Wang D Yang L Yu W Wu Q Lian J Li F. Colorectal cancer cell-derived CCL20 recruits regulatory T cells to promote chemoresistance via FOXO1/CEBPB/NF-κB signaling. J Immunother cancer (2019) 7:215. doi: 10.1186/s40425-019-0701-2 31395078 PMC6688336

[208] Guo L Zhang Y Zhang L Huang F Li J Wang S. MicroRNAs, TGF-β signaling, and the inflammatory microenvironment in cancer. Tumor Biol (2016) 37:115–25. doi: 10.1007/s13277-015-4374-2 PMC4841843 26563372

[209] Alemohammad H Najafzadeh B Asadzadeh Z Baghbanzadeh A Ghorbaninezhad F Najafzadeh A. The importance of immune checkpoints in immune monitoring: a future paradigm shift in the treatment of cancer. BioMed Pharmacother (2022) 146:112516. doi: 10.1016/j.biopha.2021.112516 34906767

[210] Peixoto A Relvas-Santos M Azevedo R Santos LL Ferreira JA. Protein glycosylation and tumor microenvironment alterations driving cancer hallmarks. Front Oncol (2019) 14:380. doi: 10.3389/fonc.2019.00380 PMC6530332 31157165

[211] Keeley TS Yang S Lau E. The diverse contributions of fucose linkages in cancer. Cancers (Basel) (2019) 11(9):1241. doi: 10.3390/cancers11091241 31450600 PMC6769556

[212] van Kooyk Y Rabinovich GA. Protein-glycan interactions in the control of innate and adaptive immune responses. Nat Immunol (2008) 9(6):593–601. doi: 10.1038/ni.f.203 18490910

[213] Radovani B Gudelj I. N-glycosylation and inflammation; the not-So-Sweet relation. Front Immunol (2022) 13:893365. doi: 10.3389/fimmu.2022.893365 35833138 PMC9272703

[214] Pereira MS Alves I Vicente M Campar. A Silva MC Padrão NA. Glycans as key checkpoints of T cell activity and function. Front Immunol (2018) 9:2754. doi: 10.3389/fimmu.2018.02754 30538706 PMC6277680

[215] Perdicchio M Cornelissen LA Streng-Ouwehand I Engels S Verstege MI Boon L. Tumor sialylation impedes T cell mediated anti-tumor responses while promoting tumor associated-regulatory T cells. Oncotarget (2016) 7(8):8771–82. doi: 10.18632/oncotarget.6822 PMC4891003 26741508

[216] Ju T Cummings RD. Protein glycosylation: chaperone mutation in tn syndrome. Nature (2005) 437(7063):1252. doi: 10.1038/4371252a 16251947

[217] RodrÍguez E Schetters STT van Kooyk Y. The tumour glyco-code as a novel immune checkpoint for immunotherapy. Nat Rev Immunol (2018) 18(3):204–11. doi: 10.1038/nri.2018.3 29398707

[218] Tam SY Wu VWC Law HKW. Hypoxia-induced epithelial-mesenchymal transition in cancers: HIF-1α and beyond. Front Oncol (2020) 8:486. doi: 10.3389/fonc.2020.00486 PMC7156534 32322559

[219] Fan H Wu Y Yu S Li X Wang A Wang S. Critical role of mTOR in regulating aerobic glycolysis in carcinogenesis (Review). Int J Oncol (2021) 58:9–19. doi: 10.3892/ijo.2020.5152 33367927

[220] Hoxhaj G Manning BD. The PI3K-AKT network at the interface of oncogenic signalling and cancer metabolism. Nat Rev Cancer (2020) 20(2):74–88. doi: 10.1038/s41568-019-0216-7 31686003 PMC7314312

[221] Hay N. Reprogramming glucose metabolism in cancer: can it be exploited for cancer therapy? Nat Rev Cancer (2016) 16:635–49. doi: 10.1038/nrc.2016.77 PMC5516800 27634447

[222] Galbraith MD Andrysik Z Pandey A Hoh M Bonner EA Hill AA. CDK8 kinase activity promotes glycolysis. Cell Rep (2017) 21(6):1495–506. doi: 10.1016/j.celrep.2017.10.058 PMC5726794 29117556

[223] Cazzaniga M Bonanni B. Relationship between metabolic reprogramming and mitochondrial activity in cancer cells. understanding the anticancer effect of metformin and its clinical implications. Anticancer Res (2015) 35:5789–96. 26503999

[224] Guerra L Bonetti L Brenner D. Metabolic modulation of immunity: a new concept in cancer immunotherapy. Cell Rep (2020) 32(1):107848. doi: 10.1016/j.celrep.2020.107848 32640218

[225] Li Q Cao L Tian Y Zhang P Ding C Lu W. Butyrate suppresses the proliferation of colorectal cancer cells via targeting pyruvate kinase M2 and metabolic reprogramming. Mol Cell Proteomics (2018) 17(8):1531–45. doi: 10.1074/mcp.RA118.000752 PMC6072541 29739823

[226] Eccleston A DeWitt N Gunter C Marte B Nath D. Epigenetics. Nature (2007) 447:395. doi: 10.1038/447395a

[227] Saleh R Toor SM Sasidharan Nair V Elkord E. Role of epigenetic modifications in inhibitory immune checkpoints in cancer development and progression. Front Immunol (2020) 11:1469. doi: 10.3389/fimmu.2020.01469 32760400 PMC7371937

[228] Dawson MA Kouzarides T. Cancer epigenetics: from mechanism to therapy. Cell (2012) 150:12–27. doi: 10.1016/j.cell.2012.06.013 22770212

[229] Singhal SK Usmani N Michiels S Metzger-Filho O Saini KS Kovalchuk O. Towards understanding the breast cancer epigenome: a comparison of genome-wide DNA methylation and gene expression data. Oncotarget (2016) 7(3):3002–17. doi: 10.18632/oncotarget.6503 PMC4823086 26657508

[230] Cheng Y He C Wang M Ma X Mo F Yang S. Targeting epigenetic regulators for cancer therapy: mechanisms and advances in clinical trials. Signal Transduction Targeting Ther (2019) 4:62. doi: 10.1038/s41392-019-0095-0 PMC6915746 31871779

[231] Newbold A Falkenberg KJ Prince HM Johnstone RW. How do tumor cells respond to HDAC inhibition? FEBS J (2016) 283:4032–46. doi: 10.1111/febs.13746 27112360

[232] Gameiro SR Malamas AS Tsang KY Ferrone S Hodge JW. Inhibitors of histone deacetylase 1 reverse the immune evasion phenotype to enhance T-cell mediated lysis of prostate and breast carcinoma cells. Oncotarget (2016) 7:7390–402. doi: 10.18632/oncotarget.7180 PMC4884926 26862729

[233] Condorelli F Gnemmi I Vallario A Genazzani AA Canonico PL. Inhibitors of histone deacetylase (HDAC) restore the p53 pathway in neuroblastoma cells. Br J Pharmacol (2008) 153:657–68. doi: 10.1038/sj.bjp.0707608 PMC2259214 18059320

[234] Sparmann A van Lohuizen M. Polycomb silencers control cell fate, development and cancer. Nat Rev Cancer (2006) 6:846–56. doi: 10.1038/nrc1991 17060944

[235] Bachmann IM Halvorsen OJ Collett K Stefansson IM Straume O Haukaas SA. EZH2 expression is associated with high proliferation rate and aggressive tumor subgroups in cutaneous melanoma and cancers of the endometrium, prostate, and breast. J Clin Oncol (2006) 24:268–73. doi: 10.1200/JCO.2005.01.5180 16330673

[236] Morin RD Johnson NA Severson TM Mungall AJ An J Goya R. Somatic mutations altering EZH2 (Tyr641) in follicular and diffuse large b-cell lymphomas of germinal-center origin. Nat Genet (2010) 42:181–5. doi: 10.1038/ng.518 PMC2850970 20081860

[237] Peng D Kryczek I Nagarsheth N Zhao L Wei S Wang W. Epigenetic silencing of TH1-type chemokines shapes tumour immunity and immunotherapy. Nature (2015) 527:249–53. doi: 10.1038/nature15520 PMC4779053 26503055

[238] Zingg D Arenas-Ramirez N Sahin D Rosalia RA Antunes AT Haeusel J. The histone methyltransferase Ezh2 controls mechanisms of adaptive resistance to tumor immunotherapy. Cell Rep (2017) 20:854–67. doi: 10.1016/j.celrep.2017.07.007 28746871

[239] Org T Chignola. F Hetényi C Gaetani M Rebane A Liiv I. The autoimmune regulator PHD finger binds to non-methylated histone H3K4 to activate gene expression. EMBO Rep (2008) 9(4):370–6. doi: 10.1038/sj.embor.2008.11 PMC2261226 18292755

[240] Abruzzese MP Bilotta MT Fionda C Zingoni A Soriani A Vulpis E. Inhibition of bromodomain and extra-terminal (BET) proteins increases NKG2D ligand MICA expression and sensitivity to NK cell-mediated cytotoxicity in multiple myeloma cells: role of cMYC-IRF4-miR-125b interplay. J Hematol Oncol (2016) 9:134. doi: 10.1186/s13045-016-0362-2 27903272 PMC5131470

[241] Shorstova T Foulkes WD Witcher M. Achieving clinical success with BET inhibitors as anti-cancer agents. Br J Cancer (2021) 124:1478–90. doi: 10.1038/s41416-021-01321-0 PMC8076232 33723398

[242] Feinberg AP. The key role of epigenetics in human disease prevention and mitigation. N Engl J Med (2018) 378:1323–34. doi: 10.1056/NEJMra1402513 PMC11567374 29617578

[243] Weyerer V Strissel PL Stöhr C Eckstein M Wach S Taubert H. Endogenous retroviral-K envelope is a novel tumor antigen and prognostic indicator of renal cell carcinoma. Front Oncol (2021) 22:657187. doi: 10.3389/fonc.2021.657187 PMC8100683 33968761

[244] Majchrzak-Celińska A Warych A Szoszkiewicz M. Novel approaches to epigenetic therapies: from drug combinations to epigenetic editing. Genes (Basel) (2021) 12(2):208. doi: 10.3390/genes12020208 33572577 PMC7911730

[245] Roulois D Loo YH Singhania R Wang Y Danesh A Shen SY. DNA-Demethylating agents target colorectal cancer cells by inducing viral mimicry by endogenous transcripts. Cell (2015) 62:961–73. doi: 10.1016/j.cell.2015.07.056 PMC4843502 26317465

[246] Emran AA Chatterjee A Rodger EJ Tiffen JC Gallagher SJ Eccles MR. Targeting DNA methylation and EZH2 activity to overcome melanoma resistance to immunotherapy. Trends Immunol (2019) 40:328–44. doi: 10.1016/j.it.2019.02.004 30853334

[247] Dobre EG Constantin C Costache M Neagu M. Interrogating epigenome toward personalized approach in cutaneous melanoma. J Pers Med (2021) 11(9):901. doi: 10.3390/jpm11090901 34575678 PMC8467841

[248] Xu Y Li P Liu Y Xin D Lei W Liang A. Epi-immunotherapy for cancers: rationales of epi-drugs in combination with immunotherapy and advances in clinical trials. Cancer Commun (Lond) (2022) 42(6):493–516. doi: 10.1002/cac2.12313 PMC9198339 35642676

[249] Bartel DP. Metazoan MicroRNAs. Cell (2018) 173:20–51. doi: 10.1016/j.cell.2018.03.006 29570994 PMC6091663

[250] Huntzinger E Izaurralde E. Gene silencing by microRNAs: contributions of translational repression and mRNA decay. Nat Rev Genet (2011) 12:99–110. doi: 10.1038/nrg2936 21245828

[251] Moles R. MicroRNAs-based therapy: a novel and promising strategy for cancer treatment. Microrna (2017) 6(2):102–9. doi: 10.2174/2211536606666170710183039 28699479

[252] Bruni D Angell HK Galon J. The immune contexture and immunoscore in cancer prognosis and therapeutic efficacy. Nat Rev Cancer (2020) 20:662–80. doi: 10.1038/s41568-020-0285-7 32753728

[253] Ziani L Chouaib S Thiery J. Alteration of the antitumor immune response by cancer-associated fibroblasts. Front Immunol (2018) 9:414. doi: 10.3389/fimmu.2018.00414 29545811 PMC5837994

[254] Monteran L Erez N. The dark side of fibroblasts: cancer-associated fibroblasts as mediators of immunosuppression in the tumor microenvironment. Front Immunol (2019) 2:1835. doi: 10.3389/fimmu.2019.01835 PMC6688105 31428105

[255] Bussard KM Mutkus L Stumpf K Gomez-Manzano C Marini FC. TumorAssociated stromal cells as key contributors to the tumor microenvironment. Breast Cancer Res (2016) 18(1):84. doi: 10.1186/s13058-016-0740-2 27515302 PMC4982339

[256] Salazar N Zabel BA. Support of tumor endothelial cells by chemokine receptors. Front Immunol (2019) 10:147. doi: 10.3389/fimmu.2019.00147 30800123 PMC6375834

[257] Maishi N Hida K. Tumor endothelial cells accelerate tumor metastasis. Cancer Sci (2017) 108(10):1921–6. doi: 10.1111/cas.13336 PMC5623747 28763139

[258] Dirat B Bochet L Dabek M Daviaud D Dauvillier S Majed B. CancerAssociated adipocytes exhibit an activated phenotype and contribute to breast cancer invasion. Cancer Res (2011) 71(7):2455–65. doi: 10.1158/0008-5472.CAN-10-3323 21459803

[259] Trivanovic D Krstic J Djordjevic IO Mojsilovic S Santibanez JF Bugarski D. The roles of mesenchymal Stromal/Stem cells in tumor microenvironment associated with inflammation. Mediat Inflamm (2016) 14. doi: 10.1155/2016/7314016 PMC5007366 27630452

[260] Akimoto N Väyrynen JP Zhao M Ugai T Fujiyoshi K Borowsky J. Desmoplastic reaction, immune cell response, and prognosis in colorectal cancer. Front Immunol (2022) 22:840198. doi: 10.3389/fimmu.2022.840198 PMC8980356 35392092

[261] Patel NR Bole M Chen C Hardin CC Kho AT Mih J. Cell elasticity determines macrophage function. PloS One (2012) 7((9):e41024. doi: 10.1371/journal.pone.0041024 23028423 PMC3445606

[262] Mun JY Leem SH Lee JH Kim HS. Dual relationship between stromal cells and immune cells in the tumor microenvironment. Front Immunol (2022) 6:864739. doi: 10.3389/fimmu.2022.864739 PMC9019709 35464435

[263] Wu L Seung E Xu L Rao E Lord DM Wei RR. Trispecific antibodies enhance the therapeutic efficacy of tumor-directed T cells through T cell receptor co-stimulation. Nat Cancer (2020) 1:86–98. doi: 10.1038/s43018-019-0004-z 35121834

[264] Runcie K Budman DR John V Seetharamu N. Bi-specific and tri-specific antibodies- the next big thing in solid tumor therapeutics. Mol Med (2018) 24:50. doi: 10.1186/s10020-018-0051-4 30249178 PMC6154901

[265] Wang H Mooney DJ. Biomaterial-assisted targeted modulation of immune cells in cancer treatment. Nat Mater (2018) 17:761–72. doi: 10.1038/s41563-018-0147-9 30104668

[266] Ngiow SF Young A. Re-education of the tumor microenvironment with targeted therapies and immunotherapies. Front Immunol (2020) 11:1633. doi: 10.3389/fimmu.2020.01633 32849557 PMC7399169

[267] Jou J Harrington KJ Zocca MB Ehrnrooth E Cohen EEW. The changing landscape of therapeutic cancer vaccines-novel platforms and neoantigen identification. Clin Cancer Res (2021) 27(3):689–703. doi: 10.1158/1078-0432.CCR-20-0245 33122346

[268] Fucikova J Hensler M Kasikova L Lanickova T Pasulka J Rakova J. An autologous dendritic cell vaccine promotes anticancer immunity in ovarian cancer patients with low mutational burden and cold tumors. Clin Cancer Res (2022) 10:4413. doi: 10.1158/1078-0432.CCR-21-4413 35536547

[269] Tornesello AL Tagliamonte M Buonaguro FM Tornesello ML Buonaguro L. Virus-like particles as preventive and therapeutic cancer vaccines. Vaccines (Basel) (2022) 10(2):227. doi: 10.3390/vaccines10020227 35214685 PMC8879290

[270] Melief CJ van Hall T Arens R Ossendorp F van der Burg SH. Therapeutic cancer vaccines. J Clin Invest (2015) 125(9):3401–12. doi: 10.1172/JCI80009 PMC4588240 26214521

[271] Riley RS June CH Langer R Mitchell MJ. Delivery technologies for cancer immunotherapy. Nat Rev Drug Discovery (2019) 18:672356. doi: 10.3389/fimmu.2021.672356 PMC6410566 30622344

[272] Wang Y Deng W Li N Neri S Sharma A Jiang W. Combining immunotherapy and radiotherapy for cancer treatment: current challenges and future directions. Front Pharmacol (2018) 9:185. doi: 10.3389/fphar.2018.00185 29556198 PMC5844965

[273] Golden EB Frances D Pellicciotta I Demaria S Barcellos-Hoff M Formenti SC. Radiation fosters dose-dependent and chemotherapy-induced immunogenic cell death. Oncoimmunology (2014) 3:e28518. doi: 10.4161/onci.28518 25071979 PMC4106151

[274] Wicki A Witzigmann D Balasubramanian V Huwyler J. Nanomedicine in cancer therapy: challenges, opportunities, and clinical applications. J Control Release (2015) 200:138–57. doi: 10.1016/j.jconrel.2014.12.030 25545217

[275] Bregoli L Movia D Gavigan-Imedio JD Lysaght J Reynolds J PrinaMello A. Nanomedicine applied to translational oncology: a future perspective on cancer treatment. Nanomed Nanotechnol Biol Med (2016) 12(1):81–103. doi: 10.1016/j.nano.2015.08.006 26370707

[276] Wang J Li S Han Y Guan J Chung S Wang C. Poly(Ethylene glycol)-polylactide micelles for cancer therapy. Front Pharmacol (2018) 8:202. doi: 10.3389/fphar.2018.00202 PMC5890116 29662450

[277] Ahmed N Fessi H Elaissari A. Theranostic applications of nano- particles in cancer. Drug Discov Today (2012) 17(17):928–34. doi: 10.1016/j.drudis.2012.03.010 22484464

[278] Martin. JD Cabral H Stylianopoulos T Jain RK. Improving cancer immunotherapy using nanomedicines: progress, opportunities and challenges. Nat Rev Clin Oncol (2020) 17:251–66. doi: 10.3390/pharmaceutics14030505 PMC8272676 32034288

